# Membrane Interactome of a Recombinant Fragment of Human Surfactant Protein D Reveals GRP78 as a Novel Binding Partner in PC3, a Metastatic Prostate Cancer Cell Line

**DOI:** 10.3389/fimmu.2020.600660

**Published:** 2021-01-19

**Authors:** Gargi Thakur, Gajanan Sathe, Indra Kundu, Barnali Biswas, Poonam Gautam, Saad Alkahtani, Susan Idicula-Thomas, Ravi Sirdeshmukh, Uday Kishore, Taruna Madan

**Affiliations:** ^1^Department of Innate Immunity, Indian Council of Medical Research (ICMR)—National Institute for Research in Reproductive Health, Mumbai, India; ^2^Institute of Bioinformatics, Bengaluru, India; ^3^Manipal Academy of Higher Education, Manipal, India; ^4^Biomedical Informatics Centre, ICMR—National Institute for Research in Reproductive Health, Mumbai, India; ^5^Laboratory of Molecular Oncology, ICMR—National Institute of Pathology, New Delhi, India; ^6^Department of Zoology, College of Science, King Saud University, Riyadh, Saudi Arabia; ^7^Biosciences, College of Health, Medicine and Life Sciences, Brunel University London, Uxbridge, United Kingdom

**Keywords:** surfactant protein D, GRP78, interactome analysis, prostate cancer, apoptosis, signaling, innate immunity

## Abstract

Surfactant protein-D (SP-D), a member of the collectin family has been shown to induce apoptosis in cancer cells. SP-D is composed of an N-terminal collagen-like domain and a calcium-dependent carbohydrate recognition domain (CRD). Recently, we reported that a recombinant fragment of human SP-D (rfhSP-D), composed of homotrimeric CRD region, induced intrinsic apoptotic pathway in prostate cancer cells. Here, we analyzed the membrane interactome of rfhSP-D in an androgen-independent prostate cancer cell line, PC3, by high resolution mass spectrometry and identified 347 proteins. Computational analysis of PPI network of this interactome in the context of prostate cancer metastasis and apoptosis revealed Glucose Regulated Protein of 78 kDa (GRP78) as an important binding partner of rfhSP-D. Docking studies suggested that rfhSP-D (CRD) bound to the substrate-binding domain of glycosylated GRP78. This was further supported by the observations that human recombinant GRP78 interfered with the binding of rfhSP-D to anti-SP-D polyclonal antibodies; GRP78 also significantly inhibited the binding of recombinant full-length human SP-D with a monoclonal antibody specific to the CRD in a dose-dependent manner. We conclude that the interaction with rfhSP-D is likely to interfere with the pro-survival signaling of GRP78.

## Introduction

Surfactant protein D (SP-D) recognizes an array of carbohydrate moieties present on the microbial surfaces (1, 2). SP-D is a hydrophilic glycoprotein; its primary structure contains N-terminal region with cysteine residues, a triple-helical collagen domain, an *α*-helical coiled-coil neck domain, and a C-terminal C-type (calcium-dependent) lectin or carbohydrate recognition domain (CRD). Four SP-D trimeric subunits assemble as a cruciform dodecamer, and their further oligomers appear as fuzzy balls under electron microscope (3–5). Pathogens bound to SP-D get aggregated and opsonized leading to enhanced phagocytosis and oxidative damage (6). The CRD of SP-D interacts with various self-ligands as well as receptors such as CD14, Signal Inhibitory Regulator protein (SIRP)-*α*, Toll-like receptor (TLR)-2, and TLR-4 to bring about immune regulation (7, 8).

An important role of SP-D in allergy was noted when a recombinant fragment of human SP-D (rfhSP-D) composed of neck and CRD region induced apoptosis in activated eosinophils of allergic patients (9); this involved p53 upregulation as demonstrated in rfhSP-D treated AML14.3D10 cells, an eosinophilic leukemic cell line (10). Subsequently, SP-D was shown to bind the Epidermal Growth Factor Receptor (EGFR) on A549 lung cancer cells, inducing cell death (11). Kaur et al. recently reported that rfhSP-D induced apoptosis in pancreatic cancer cell lines *via* TNF-α/Fas pathway irrespective of the p53 status (12). Further, inhibition of TGF-*β* expression in the pancreatic cancer cells suppressed their epithelial-to-mesenchymal transition (EMT) and ability to invade (13). A significantly reduced expression of SP-D transcripts has been reported in lung, gastric, and breast cancers, whereas ovarian cancer tissues express more SP-D. The presence of SP-D predicted a favorable outcome in lung cancer. Conversely, in gastric, breast, and ovarian cancers, SP-D expression suggested a poor prognosis (14).

Reduced expression of SP-D has been observed in the glandular cells of human prostate cancer tissues. SP-D levels negatively correlated with Gleason score, a scoring system based on the cancer cell types and patterns on the tissue sections as well as tumor volume (15). We recently reported that androgens regulate SP-D expression in the androgen-sensitive LNCaP prostate adenocarcinoma cells (16). Importantly, rfhSP-D specifically induced apoptosis in the tissue explants, primary tumor cells of patients with metastasized prostate cancer, and the prostate cancer cell lines (LNCaP, PC3). The rfhSP-D treatment did not affect the normal prostate epithelial cells (16).

The broad spectrum anti-cancer activity of rfhSP-D is possibly due to the simultaneous targeting of multiple growth factors, kinases, transcription factors, and apoptotic pathways (9–13, 16). Here, we set out to examine the interaction of rfhSP-D with the membrane proteins of the prostate cancer cell line, PC3, using Mass Spectrometry-based LC-MS/MS analysis. Based on the protein−protein interaction (PPI) network analysis of the interactome proteins, Glucose Regulated Protein of 78 kDa (GRP78) was found to be an important interactor and was thus selected for further analysis.

GRP78 is an endoplasmic reticulum (ER) resident chaperone and is also known as HSPA5. GRP78 acts as an ER stress sensor and is upregulated under stress conditions, for maintaining ER homeostasis and cell survival. For cellular homeostasis, GRP78 interacts with three ER stress sensor proteins: protein kinase RNA-like ER kinase (PERK), inositol-requiring kinase 1 (IRE1*α*), and activating transcription factor 6 (ATF6). However, under ER stress conditions, unfolded proteins titrate away GRP78 to activate the unfolded protein response (UPR) and alleviate cellular stress. Activated UPR decreases protein influx into the ER and induces the synthesis of components involved in protein folding to support cell survival (17). In addition, GRP78 localizes to cell surface (csGRP78) and operates as a receptor, interacting with various ligands to induce pro-proliferative, pro-survival, and pro-apoptotic signalling (18). GRP78 is significantly upregulated in various cancers due to over stressful microenvironment (17). The upregulated expression of GRP78 in patients with castration-resistant prostate cancer has been associated with resistance to chemotherapy (19, 20). Here, using mass spectrometry, protein-protein interactome analysis and molecular modelling, we report GRP78 as a putative receptor for the CRD region of human SP-D. The interaction between rfhSP-D and GRP78 may interfere with pro-tumorigenic role of GRP78 in prostate cancer and could be a plausible mechanism for rfhSP-D-mediated apoptosis of prostate cancer cells.

## Materials and Methods

### Cell Culture

Human prostate cancer cell line, PC3 (androgen independent, p53^-/-^) that mimics biochemical changes in advanced prostate cancer, has been significantly responsive to rfhSP-D induced apoptosis (16). PC3 cells (ATCC, Rockville, MD, USA) were grown in RPMI 1640 supplemented with 10% v/v Foetal Bovine Serum (FBS) and 1% Antibiotics (PenStrep). To achieve approximately 90% confluence, PC3 cells were incubated in 5% v/v CO_2_ at 37°C.

### Purification of rfhSP-D

The rfhSP-D (179–355 residues comprised of a functional homotrimeric lectin domain (236–355 aa residues), followed by the neck region (203–235 aa residues) and eight Gly-X-Y repeats from the collagen domain (179–202 aa residues). Using *Escherichia coli* BL21 (*λ*DE3) pLysS (Invitrogen), rfhSP-D was expressed and purified as described earlier (9). The QCL-1000 LAL system (Bio Whittaker Inc., USA) was used to assess the endotoxin levels. Linearity of the assay was observed in the range of 0.1–1.0 EU/ml or 0.01–0.1 ng/ml endotoxin. Purified rfhSP-D contained <4 pg of endotoxin per µg of rfhSP-D.

### Isolation of PC3 Membrane Proteins Interacting With rfhSP-D

PC3 cells (5 × 10^6^/ml) were pre-incubated with in serum-free RPMI medium containing rfhSP-D (20 µg/ml) and 5 mM CaCl_2_ for 2 h at 37°C in the CO_2_ incubator (PC3 cells were pre-incubated with rfhSP-D to enable isolation of complexes of rfhSP-D and membrane proteins). Following incubation, the unbound rfhSP-D was removed along with the medium followed by a wash with sterile PBS. Then, PC3 cells were harvested for membrane protein isolation by scrapping in the wash solution supplied with Mem-PER™ Plus Membrane Protein Extraction Kit (Thermo Fisher Scientific; #89842).

For the co-immunoprecipitation and pull-down experiments, 10 µg of membrane protein extract, along with bound rfhSP-D, was combined with 10 µg of polyclonal antibody against human SP-D (Santacruz) and incubated overnight at 4°C with mixing. First, Pierce Protein A/G Magnetic beads (Thermo Fisher Scientific; #88802) were washed three times with TBST wash buffer. The antigen and antibody mixture was added to 1.5 ml tube containing pre-washed protein A/G magnetic beads (250 µg) and incubated at room temperature for 2 h on a rotary shaker. Beads were then separated on a magnetic stand, and the flow-through was saved for analysis. Beads were washed three times with the wash buffer and a final wash was given with Milli Q water. Beads were then resuspended in the elution buffer (0.1 M glycine, pH 2.0) and incubated at room temperature for 10 min with mixing. Beads were separated magnetically and supernatant containing the target antigen was saved for further analysis. Low pH was neutralized by adding neutralizing buffer (1M Tris-HCl, pH 7.5) to the eluate. PC3-derived membrane protein fraction used for LC-MS/MS analysis was pooled from five independent pull-down experiments with three technical replicates.

### Western Blot Analysis of Various Fractions

PC3 cells (5 × 10^6^) in serum-free RPMI medium were plated in a T75 tissue culture flask and pre-incubated with rfhSP-D (20 μg/ml) for 2 h, as described above. PC3-membrane as well as cytoplasmic proteins were isolated, as described above, and analyzed by Western blotting. Lysate proteins (30 μg) were subjected to 12% v/v SDS-PAGE and electro-transferred onto PVDF membranes (Pall Corporation, NY, USA). To confirm the presence of rfhSP-D binding proteins in the two protein fractions, one of the blot was blocked (3% skimmed milk powder in TBS, 45 min on a shaker at room temperature), incubated with rfhSP-D in the presence of CaCl_2_ or EDTA (2 h at 37°C), and probed with primary rabbit anti-human SP-D polyclonal conjugate (overnight at 4°C) and secondary goat anti-rabbit Horseradish Peroxidase (HRP) conjugate (1 h at room temperature on a shaker). To confirm the purity of the two protein fractions, another blot was probed with primary antibodies against human Prostate-Specific Membrane Antigen (specific to the membrane protein fraction of prostate cells) (rabbit monoclonal PSMA antibody; Cell Signalling Technology, # D7I8E, 1:500) overnight at 4°C, followed by HRP-conjugated secondary goat anti-rabbit IgG antibodies (diluted 1:6000) (1 h at room temperature on a shaker). The blots were developed using the enhanced chemiluminescent (ECL) Kit (Millipore, USA). The blot images were captured using the Syngene (Chem Genius).

### Immune-Depletion of Proteins From Membrane Fraction

Membrane proteins in the eluate were concentrated using 3 K filters (Millipore, MA, USA) and then subjected to IgG depletion using Multiple Affinity Removal System Spin Cartridge, HSA/IgG (Agilent, CA, USA), following the manufacturer’s instruction. A buffer exchange followed using Amicon Ultra-0.5 (3K) device in PBS. Total protein was estimated by Bradford assay, followed by SDS-PAGE analysis to assess the profile of cell lysate, and membrane fraction with rfhSP-D treatment after immune-depletion.

To confirm the presence of rfhSP-D in the two protein fractions (20 μg), the supernatant of pull-down fraction (5 μg), flow-through of pull-down fraction (5 μg), eluate of pull-down (5 μg), and flow-through containing IgG after immune-depletion (5 μg) were separated *via* 12% v/v SDS-PAGE and electro-transferred to PVDF membranes. Then, the membrane was blocked and incubated with the rabbit anti-human SP-D polyclonal antibodies (overnight at 4°C) and secondary goat anti-rabbit HRP conjugate (1 h at room temperature). The blot was developed using the enhanced chemiluminescent (ECL) Kit (Millipore, USA). Image was acquired by Syngene (Chem Genius).

### Lys-C/Trypsin Digestion

Eluates of the five individual pull-down experiments were diluted in ammonium bicarbonate buffer (TEABC; Sigma) to a final volume of 100 μl, followed by reduction using 10 mM dithiothreitol (DTT) for 20 min at 60°C. Once the proteins came to room temperature, they were alkylated (20 mM Iodoacetamide; Merck) for 10–15 min at room temperature in the dark. The proteins were digested with 1 μg of Lys-C for 3–4 h at room temperature, followed by digestion with 2 μg of trypsin overnight at 37°C. Enzyme activity was terminated with 1% v/v formic acid. Peptides were fractionated *via* C-18 columns and eluted with a buffer comprising acetonitrile (40%) and formic acid (0.1%). Fractionated peptides were lyophilized on a SpeedVac (30 min at 45°C) and then solubilized in 0.1% formic acid prior to LC-MS/MS analysis.

### Mass Spectrometry

Orbitrap Fusion Tribrid mass spectrometer (Thermo Fisher Scientific), connected with an Easy-nLC II nanoflow liquid chromatography system (Thermo Fisher Scientific), was used for peptide analysis. A trap column (75 µm × 2 cm, Magic-C18-AQ material 5 µm, 100 Å) was used to enrich peptides. The peptides were separated at a flow rate of 30 ml/min on a 20 cm long column of 5 µm Magic-C18-AQ (Michrom Bioresources, Inc., Auburn, CA, USA) using a gradient of 8–30% solvent B (90% acetonitrile in 0.1% formic acid) over 103 min for a 120 min run. Mass spectrometry data were collected at a resolution of 120,000 in a range of 350–1,600 m/z. The highly intense ions with charge state >2 were isolated in 3 s cycle and subjected to HCD fragmentation with 32% normalized collision energy. These fragmented ions were sensed at a resolution of 15,000 at 200 m/z. The limit of dynamic exclusion was fixed at 40 s with a 10-ppm mass window. The maximum ion injection times were 50 ms for MS and 75 ms for MS/MS. The automatic gain control targets were 4 × 10^5^ for MS and 1 × 10^5^ for MS/MS.

### Identification of Peptides and Proteins From Database

The uninterpreted MS/MS data from the complete LC-MS/MS run was subjected to a search in the database (Human RefSeq protein database). The search algorithm used was SEQUEST, and the platform used was Proteome Discoverer (version 2.1, Thermo Scientific). A maximum of two missed cleavages, carbamidomethylation at cysteine as fixed and oxidation of methionine as variable modifications, were included as the search parameters. Mass tolerance of monoisotopic peptide was set at 10 ppm and the MS/MS tolerance was limited to 0.02 Da. At the PSM as well as the protein level, the false discovery rate of 1% was set. MS data has been submitted to the ProteomeXchange Consortium (http://www.proteomexchange.org) *via* the PRIDE partner repository (dataset identifier PXD008098).

### Ingenuity Pathway Analysis

Proteins identified by LC-MS/MS analysis were further analyzed for their interactions and molecular pathways using Ingenuity Pathways Analysis (IPA) software (http://www.ingenuity.com) (21).

### Network Analysis

Protein–protein interactions (PPIs) of the rfhSP-D interactome (347 proteins) were downloaded from STRING v11 (22). Proteins in the network were scored based on network topological properties, such as degree of connectivity, clustering coefficient, betweenness centrality, closeness centrality, and shortest path using R package igraph (23). The importance of each protein in the network was assessed based on perturbation and disruption scores. Perturbation score for each protein reflected the difference in centrality of network, calculated based on average of clustering coefficient, betweenness centrality, and closeness centrality after deletion of a protein (24). Disruption score of each protein was calculated based on average increase in shortest path length of protein pairs caused by deletion of the protein (25). Proteins were ranked individually based on hub (degree of connectivity), perturbation and disruption analysis; the average rank obtained from the three methods was used for creating the final ranked list. The top 5% proteins of the rank list were shortlisted and screened for their expression in prostate glandular cells as per protein atlas data (26); prostate cancer (C0376358) and metastasis of prostate cancer (C1282496) using data available in DisGeNET (27); and role in cancer cell apoptosis and survival as per information in ApocanD database (28) and IPA.

### Protein Docking

The crystal structure of human GRP78 complexed with ADP (produced in *Escherichia coli* BL21) (PDB ID: 5E84 Chain A) was downloaded from PDB (29). O-glycosylation of GRP78 is critical for its stability, subcellular localization, and anti-apoptotic function (30). Putative O-glycosylation sites have been identified in GRP78 (Thr85, Thr151, Thr166, Thr184, and Thr203) based on *in vitro* immunoprecipitation assays (30). The crystal structure of GRP78 was glycosylated by addition of five O-linked N-acetyl-galactosamine (GalNAc) at the putative sites. The glycosylated structure was generated using CHARMM-GUI web server and optimized using CHARMm force field (31). Monomeric form of GRP78 is present in stress induced cells (32); hence, the monomeric glycosylated structure of GRP78 was blind docked with crystal structure of CRD domain of active recombinant fragment of human lung surfactant protein SP-D (PDB ID:1PW9) using ZDOCK (Biovia, Discovery studio Version 2017) (33, 34). The input parameters for docking were set as default options. The top 2,000 docked poses generated by ZDock were re-ranked utilizing detailed electrostatic, van der Waals and solvation forces by ZRank (35, 36). The top ranked pose based on ZRank was further analyzed for intermolecular interactions.

### Interaction Studies between rfhSP-D/rFLhSP-D and GRP78 by ELISA

To analyze the rfhSP-D/rFLhSP-D (recombinant full-length human SP-D; R & D) interaction with GRP78, the study utilized non-glycosylated version of GRP78 (purified from *E. coli*; Cayman, catalog no. 22730) since the O-linked glycosylated GRP78 specific to cancer cells is a minor fraction of the total GRP78 (30). Using a direct ELISA, GRP78 (1 µg/ml in PBS) was coated on microtitre wells overnight at room temperature. Post-washing (three times with PBST, PBS + 0.05% Tween 20), the wells were incubated with 1% w/v BSA in PBS for 1 h at room temperature to block the additional sites. After extensive washing, rfhSP-D (1 µg/ml in PBS buffer) was added to the wells with and without 5 mM CaCl_2_, or EDTA (10 mM). Bound rfhSP-D was probed with primary rabbit anti-human SP-D polyclonal antibodies (1 h at 37°C) and secondary goat anti-rabbit HRP conjugate (1 h at 37°C), or with biotinylated Human SP-D detection antibody (500 ng/ml; Duoset SP-D ELISA kit, R & D Systems, USA) (1 h at room temperature with shaking) and Streptavidin-HRP conjugate (1:200; 45 min at room temperature with shaking).

The ability of GRP78 to bind rfhSP-D and block its binding to rabbit anti-SP-D polyclonal antibodies was analyzed by coating rfhSP-D (1 µg/ml in PBS) in duplicates, followed by blocking and incubation with GRP78 (1 µg/ml in PBS). The wells were then probed with primary rabbit anti-human SP-D polyclonal antibodies and secondary goat anti-rabbit HRP conjugate.

The ability of GRP78 to bind rFLhSP-D and block its binding to anti-SP-D monoclonal antibody was analyzed by a sandwich ELISA (Human SP-D Duoset ELISA kit). Human SP-D capture antibody was coated (2 µg/ml) overnight at room temperature. After washing, the wells were incubated with 1% w/v BSA in PBS for 1 h at room temperature to block the unoccupied sites. The wells were then incubated with 5 ng/ml of rFLhSP-D (116 × 10^−3^ nM) and increasing concentrations of human recombinant GRP78 from 5 ng/ml (70 × 10^−3^ nM) up to 20 ng/ml (280 × 10^−3^ nM) (1 h at 37°C), and probed with Human SP-D detection antibody (500 ng/ml) (1 h at room temperature with shaking). After washing, the wells were incubated with Streptavidin-HRP conjugate (1:200) (45 min at room temperature). Color was developed using a substrate solution [1:1 mixture of Color Reagent A (H_2_O_2_) and Color Reagent B (Tetramethylbenzidine); optical density was measured at 450 nm using an ELISA plate reader (Beckman Coulter).

### Statistical Analysis

The data obtained for various experiments were analyzed for statistical significance and graphical representation using GraphPad PRISM ver6.0 (GraphPad Software Inc., San Diego, CA). To compare the untreated and treated groups, the unpaired t-test was used. Data have been represented as mean ± SD. The p values less than 0.05 showed that the findings were statistically significant.

## Results

### Isolation of Membrane Proteins From rfhSP-D Pre-Treated PC3 Cells

**Figure 1:**
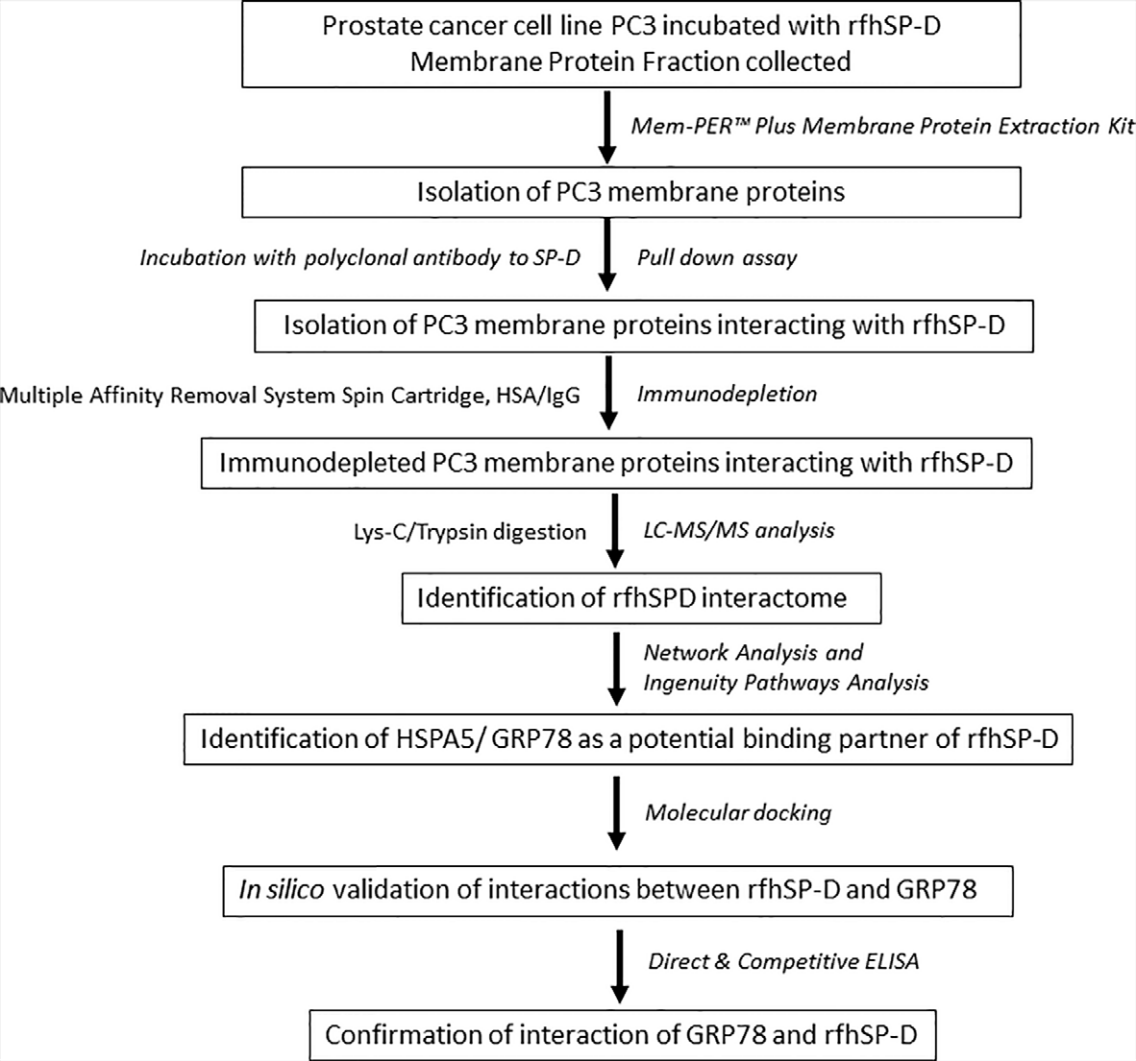
Work flow and data analysis. PC3 cells were incubated with rfhSP-D for 2 h at 37°C. Cells were then subjected to membrane protein extraction. The isolated PC3 cell membrane proteins were incubated with polyclonal anti-human SP-D antibody for obtaining rfhSP-D-bound membrane proteins (pull-down assay). The rfhSP-D interacting membrane proteins were subjected to immunodepletion. The immunodepleted rfhSP-D-bound PC3 cell membrane proteins were subjected to LC-MS/MS analysis. The identified rfhSP-D interacting membrane proteins were further examined using Network and Ingenuity Pathways Analysis. HSPA5/GRP78 was identified as a potential binding/interacting partner of rfhSP-D and was confirmed by *in silico* analysis (molecular docking) and *in vitro* studies (Direct and competitive ELISA).

To investigate the rfhSP-D interactome in PC3 cells, we performed co-immunoprecipitation and pull-down assays using polyclonal antibodies to rFLhSP-D, followed by immune-depletion of abundant proteins (HSA/IgG) and LC-MS/MS analysis (workflow scheme shown in **Figure 1**).

**Figure 2:**
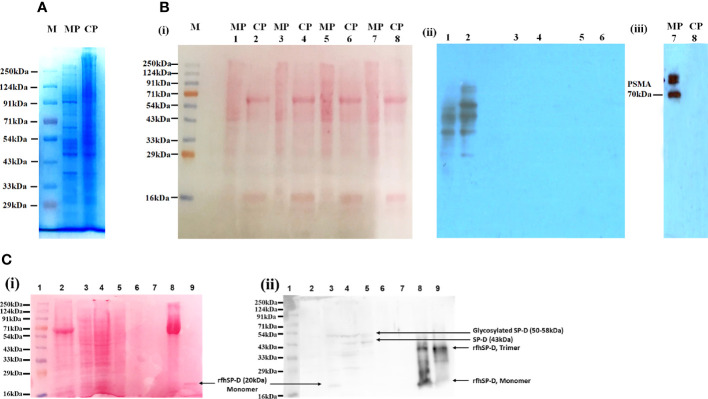
Isolation and characterization of the proteins in PC3 cell lysate. **(A)** Visualization of isolated protein fractions. Membrane Protein (PC3-M) and Cytosolic Protein (PC3-C) from PC3 cells by Coomassie Brilliant Blue dye gel staining. **(B)** Presence of rfhSP-D interacting partners in the isolated protein fractions PC3-C and PC3-M (Far-Western/ligand blotting). (i) electro-transferred to PVDF membrane stained with Ponceau dye used as loading control. (ii) Lanes 1, 2: Incubated with rfhSP-D and CaCl_2_; Lane 3s, 4: Incubated with rfhSP-D, CaCl_2_ and EDTA; Lanes 5, 6: Incubated with Secondary goat anti-rabbit HRP conjugate alone (antibody control). (iii) Prostate specific Membrane Antigen (PSMA) specifically expressed in isolated PC3 cells membrane protein fraction. **(C)** rfhSP-D was not detected in the immune-depleted, rfhSP-D bound fraction of PC3-M and was present in the IgG/HSA and interacting proteins enriched eluate of rfhSP-D bound fraction of PC3-M. (i) Various electrophoresed protein fractions were electro-transferred to PVDF membrane and stained with Ponceau dye to reveal the profile. (ii) Lane 1: Ladder; Lane 2: Entire PC3 cell protein (PC3) (20 μg); Lane 3: Cytosolic protein fraction of rfhSP-D treated PC3 cell (PC3-C) (20 μg); Lane 4: Membrane protein fraction of rfhSP-D treated PC3 cell (PC3-M) (20 μg); Lane 5: rfhSP-D bound fraction of PC3-M (7 μg); Lane 6: rfhSP-D unbound fraction of PC3-M (5 μg); Lane 7: Immune-depleted rfhSP-D bound fraction of PC3-M (flow-through of Multiple Affinity Removal System Spin Cartridge, HSA/IgG) (5 μg); Lane 8: IgG/HSA and bound proteins enriched eluate of rfhSP-D bound fraction of PC3-M (Eluate from Multiple Affinity Removal System Spin Cartridge, HSA/IgG) (20 μg); Lane 9: rfhSP-D (0.5 μg). Expected molecular weight of monomeric rfhSP-D is approximately 20 kDa, and it can exist as a dimer and trimer. MW of native SP-D is 43 kDa under reducing conditions, and glycosylated forms of SP-D are with an mw of 50–58kDa. Proteolytically truncated forms of native SP-D appear at ~25kDa.

The protein profile of the membrane as well as cytosolic fractions isolated after rfhSP-D pull-down assay using rfhSP-D-treated PC3 cells was distinct, as revealed by SDS-PAGE (**Figure 2A**). **Figures 2B(i)** and **C(i)** represent protein bands electro-transferred to PVDF membrane, stained with Ponceau dye and used as loading control. Ligand blotting of the membrane and cytosolic protein fractions with rfhSP-D showed presence of rfhSP-D binding proteins in both the fractions [**Figure 2B(ii)**]. Western blot analysis showed significant enrichment of Prostate-specific Membrane Antigen (PSMA) in the membrane fraction in comparison to cytosolic fraction [**Figure 2B(iii)**]. **Figure 2C(ii)** shows the Western blot of isolated PC3 cell membrane proteins after 2 h incubation with rfhSP-D and subsequent fractions, using anti-human SP-D polyclonal antibody. Following 2 h incubation, the unbound rfhSP-D was removed along with the medium, followed by a wash with sterile PBS. Hence, only the ligand-bound rfhSP-D is expected to be present in the protein fractions analyzed. Native SP-D protein levels in the cell culture supernatants of PC3 cells (1 × 10^6^/ml–3.2 µg of total protein/µl), as evaluated by ELISA, were found to be 220 ± 21 pg/ml, indicating approximately 68.75 pg of SP-D/mg of protein (16). Expected molecular weight of rfhSP-D is approximately 20 kDa and that of full-length SP-D is ~43 kDa under reducing conditions. Both the native full-length SP-D and rfhSP-D are known to exist as oligomers; post-translationally modified and proteolytically truncated forms of native SP-D have also been reported (37–39). Therefore, multiple bands representing various forms of native human SP-D from the PC3 cells and ligand-associated rfhSP-D could be seen in the Western blot.

The lanes 2, 3, 4 are PC3 cell entire protein (PC3), cytosolic (PC3-C), and membrane protein (PC3-M) (20 µg). Owing to the low amounts of native SP-D and rfhSP-D in the cell lysate and fractions, the anti-human SP-D antibody could detect multiple faint bands, suggesting presence of different oligomeric and truncated forms. Lane 5 was anti-human SP-D antibody pull-down eluate (rfhSP-D-bound fraction of PC3-M) (7 µg) that showed the presence of oligomeric forms of SP-D and rfhSP-D. Lane 6 was the supernatant of pull-down eluate (rfhSP-D- unbound fraction of PC3-M) (5 µg), and therefore, was expected to show minimal amounts of rfhSP-D. Lane 7 (5 µg) was the immune-depleted, rfhSP-D-bound fraction of PC3-M (flow-through of Multiple Affinity Removal System Spin Cartridge, HSA/IgG) that showed the absence of native SP-D and rfhSP-D. Native SP-D and rfhSP-D can bind various classes of immunoglobulins, including IgG, IgM, IgE and secretory IgA, *via* its CRD region in a calcium-dependent manner (40). Thus, after immune-depletion of abundant proteins IgG/HSA and interacting proteins (including rfhSP-D and native SP-D) from the rfhSP-D-bound fraction of PC3-M, the flow-through did not contain rfhSP-D and native SP-D. This was further corroborated by the LC-MS/MS analysis of this fraction wherein the native SP-D and rfhSP-D were not detected. It is important to note that some of the rfhSP-D-binding proteins may have also been missed out in the pull-down eluate. Lane 8 is the IgG/HSA and interacting proteins enriched eluate of rfhSP-D-bound fraction of PC3-M (eluate from Multiple Affinity Removal System Spin Cartridge, HSA/IgG) (20 µg). This fraction, as expected, showed different oligomeric and truncated forms of native SP-D and rfhSP-D. Lane 9 was purified rfhSP-D (0.5 µg) loaded as a positive control.

**Figure 3:**
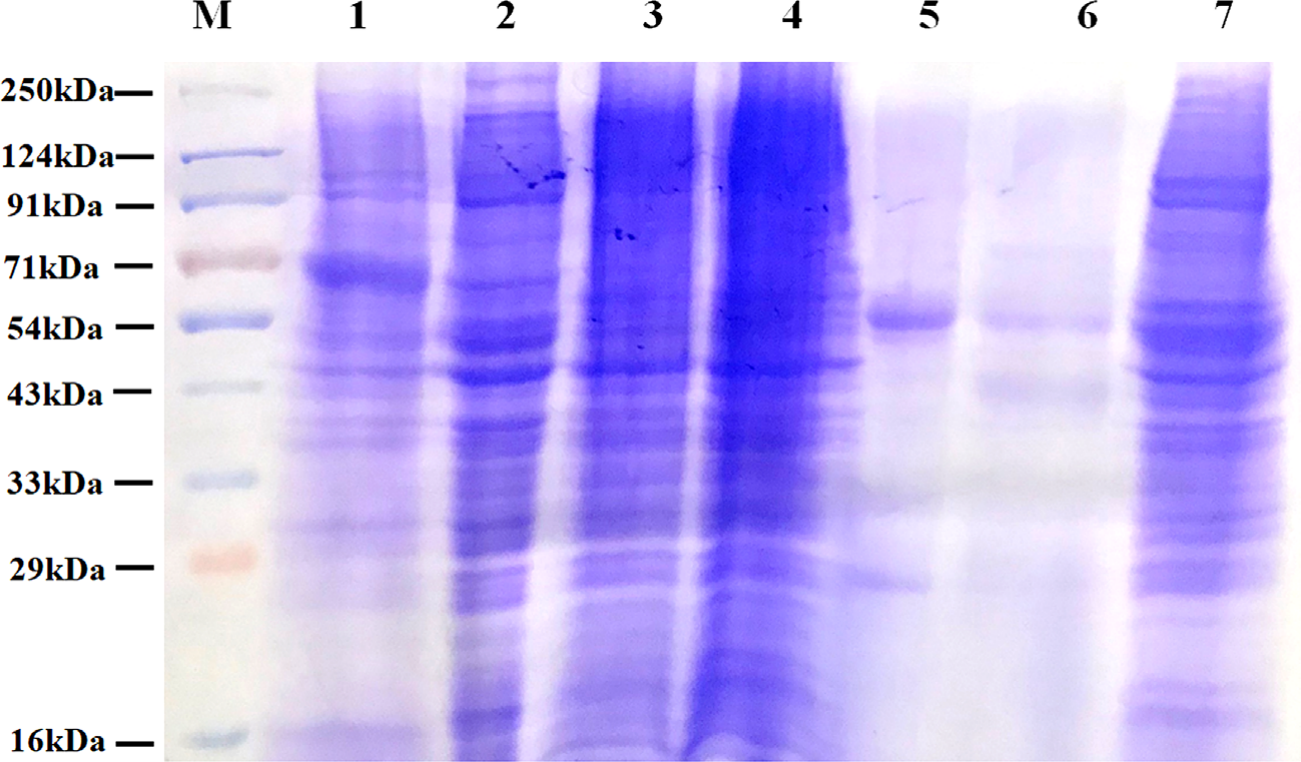
Protein profile of different fractions of PC3 cells before and after co-immunoprecipitation. Lane 1: Total PC3 cell protein (PC3, 15 µg); Lane 2: Cytosolic protein fraction of rfhSP-D treated PC3 cells (PC3-C, 20 µg); Lane 3: Membrane protein fraction of PC3 cells (20 µg); Lane 4: Membrane protein fraction of rfhSP-D treated PC3 cells (PC3-M, 20 µg); Lane 5: rfhSP-D bound fraction of PC3-M (5 µg); Lane 6: Immune-depleted rfhSP-D bound fraction of PC3-M (7 µg); Lane 7: IgG/HSA and bound proteins enriched eluate of rfhSP-D bound fraction of PC3-M (20 µg).

IgG and other abundant proteins in the membrane fraction were immune-depleted using Multiple Affinity Removal Spin Cartridge HSA/IgG as they can potentially mask the detection of low abundant proteins on LC-MS/MS analysis. SDS-PAGE profile showed enrichment of other proteins following immune-depletion of IgG and HSA (**Figure 3**).

### Identification of the Membrane Proteins of PC3 Cells Interacting With rfhSP-D

Mass spectrometric analysis (LC-MS/MS) of PC3 cell membrane fraction, treated with rfhSP-D, led to the identification of a total of 672 proteins, out of which 347 proteins were detected with ≥2 unique peptides, each with at least two peptide-spectrum matches (PSMs) and were considered as rfhSP-D interactome (**Supplementary File S1**). For each protein, accession, gene symbol, description, protein name, molecule weight, sequence coverage (%), PSMs, unique peptides and Score Sequest HT: Sequest HT have been provided (**Supplementary File S2**). Based on the PSM values from LC-MS/MS data, GRP78 (HSPA5; PSM value: 72) was the top ranked protein in the list of 347 proteins identified in the rfhSP-D interactome. Interestingly, six more members of the heat-shock protein (HSP) family that are reported to be involved in regulation of apoptosis of cancer cells, namely HSP90AB1 (PSM value: 55), HSPA8 (PSM value: 54), HSP90AA1 (PSM value: 54), HSPA1B (PSM value: 39), HSP90B1 (PSM value: 36), and HSPD1 (PSM value: 35), were listed among the first fifteen proteins. Some of the reported SP-D receptors such as DEFA1 (Human Alpha Defensin 1), CALR (Calreticulin), C1QBP (C1q receptor, gC1qR), and A2ML1 (*α*2-macroglobulin-like protein 1), were also present in the identified interactome (**Supplementary File S1**) (2, 6). Identification of these putative SP-D receptors validated the interactome analysis. As discussed above, the proteins identified from the LC-MS/MS analysis did not include SP-D. Therefore, SP-D was added to the list of 347 proteins for subsequent bioinformatics analysis in order to decipher the networks and protein–protein interactions.

### Pathway Analysis of the rfhSP-D Interactome

**Figure 4:**
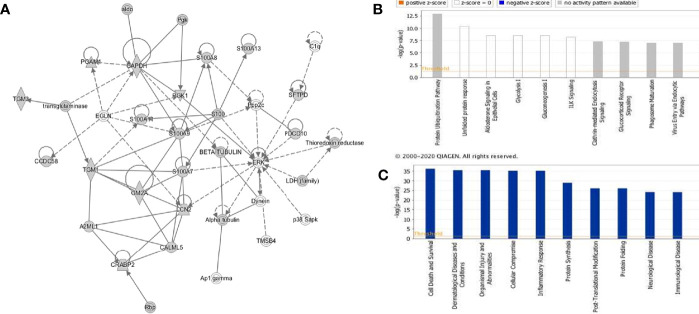
Ingenuity pathway analysis-based rfhSP-D networks in metastatic prostate cancer cells. **(A)** Protein network obtained using Ingenuity Pathway Analysis (IPA) demonstrating interactions of rfhSP-D with proteins involved in the Cellular Compromise, Dermatological Diseases and Conditions, Organismal Injury and Abnormalities. **(B)** Canonical Pathway analysis categorizing rfhSP-D interacting proteins into different pathways such as Protein Ubiquitination Pathway, unfolded protein response, Aldosterone Signaling, Glycolysis I, and Gluconeogenesis (I) **(C)** Upstream analysis of rfhSP-D interactome segregated proteins in the following pathways—Cell Death and Survival, Cellular Compromise, Protein Synthesis, Post-Translational Modification, and Protein Folding.

The rfhSP-D interactome [348 proteins, 347 identified proteins + SP-D (P35247)] was analyzed by ingenuity pathway analysis (IPA) for associated biological functions, canonical pathways, and networks. Our network analysis revealed novel signaling proteins that are likely to interact (directly/indirectly) with rfhSP-D and/or regulate its associated networks (**Figure 4A**). The binding partners of rfhSP-D identified herein were also classified on the basis of their molecular and cellular functions (**Figure 4C**). Cell Death and Survival, Cellular Compromise, Protein Synthesis, Post-Translational Modification and Protein Folding emerged as significant categories in the rfhSP-D binding proteins. IPA revealed that top five canonical signaling pathways significantly associated with rfhSP-D (p-value < 0.001; **Figure 4B**). Protein Ubiquitination Pathway was the top-ranked (p value = 1.26E-13) canonical pathway that included GRP78 (HSPA5) and SFTPD.

IPA analysis further revealed the involvement of several transcription factors such as FOXM1, JUND, FOXA2, JUNB, CEBPA, JUN, CEBPB, and FOS that regulate the expression of SP-D. The human SP-D gene promoter has a conserved activator protein-1 (AP-1) element (−109) wherein transcription factors of the *fos* and *jun* families bind (41). Foxm1 regulates transcription of several genes involved in the surfactant homeostasis and lung development, including all the four surfactant-associated proteins-SP-A, SP-B, SP-C, and SP-D (42). CCAAT-enhancer-binding protein (C/EBP) transcription factors are required for basal and enhanced SP-D promoter activity, as evident in C/EBP*β* cDNA co-transfected H441 cells (43).

### Network Analysis of rfhSP-D Interactome

**Figure 5:**
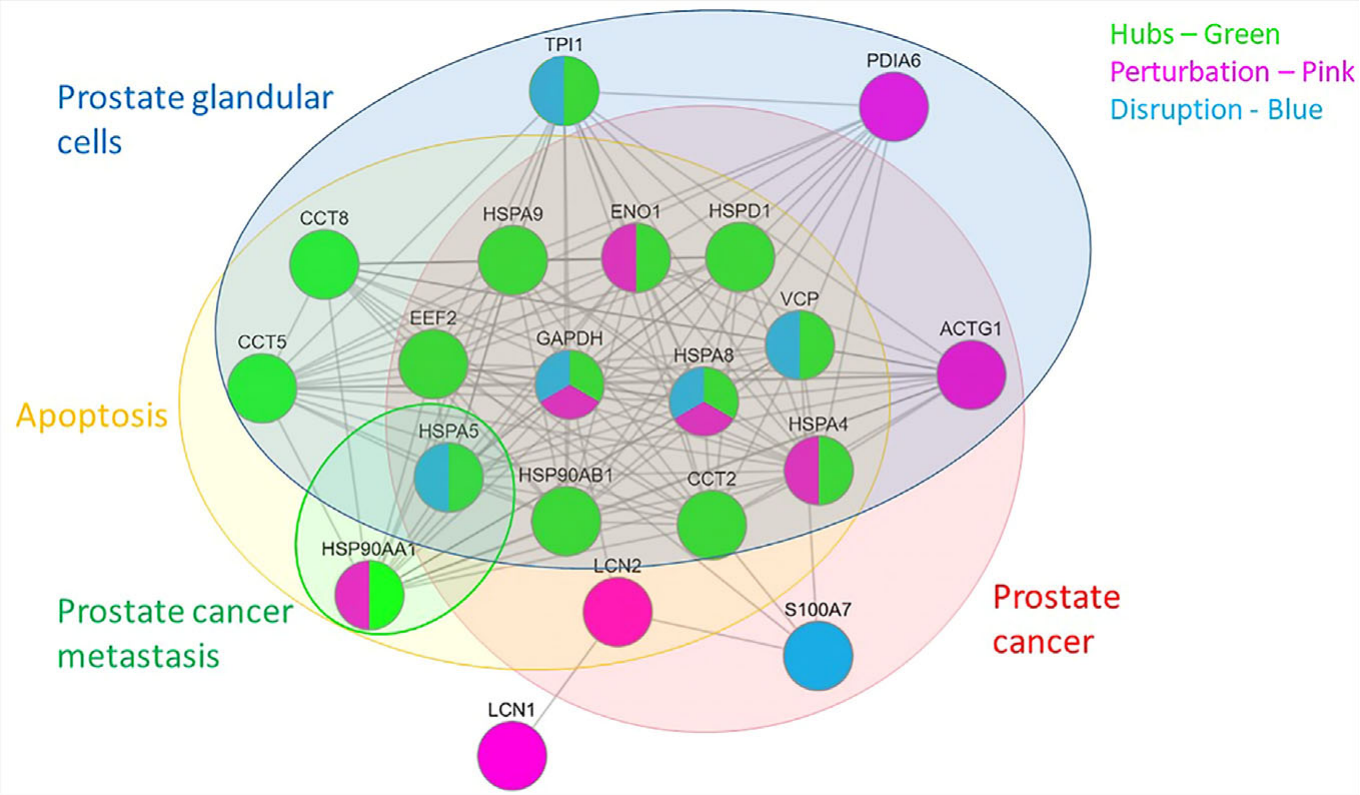
Network analysis of rfhSP-D binding proteins of PC3 cells. Network representation of 20 shortlisted proteins from network analysis; pie of node represents the analysis (hub, perturbation, disruption). Overlaying Venn represents proteins known to be associated with prostate cancer, prostate cancer metastasis, apoptosis, and expressed in prostate cells.

The PPI network of 347 proteins was analyzed for network parameters (*Materials and Methods*) to shortlist critical proteins of the rfhSP-D interactome. 20 proteins (GAPDH, HSPA8, HSP90AA1, HSPA4, TPI1, ENO1, VCP, CCT2, HSPA5, LCN2, EEF2, ACTG1, HSPD1, PDIA6, CCT8, S100A7, HSPA9, LCN1, HSP90AB1, CCT5) were selected and ranked based on connectivity, perturbation, and disruption scores (**Table 1**; **Supplementary File S3**). 15 of these 20 proteins are known to be associated with prostate cancer, 13 with prostate cancer as well as apoptosis, and only two (HSP90AA1, HSPA5) are known to be associated with metastasis of prostate cancer (**Figure 5**; **Table 2**; **Supplementary File S2**). Of the two, HSPA5 is known to be expressed in prostate glandular cells as per the protein atlas; therefore, it was selected for further *in silico* and *in vitro* interaction studies. A summary of the network analysis of rfhSP-D interactome is represented as a Venn diagram (**Figure 5**). HSPA5 or GRP78, a chaperone expressed on cell surface of cancer cells only, is known to be associated with malignancy, development of castration-resistant prostate cancer, and resistance to chemotherapy. GRP78 seems to act upstream of PI3K/Akt; monoclonal antibody against GRP78 suppresses pAkt expression, suggesting promotion of apoptosis (44).

**Table 1:** Shortlisted proteins from the rfhSP-D interactome and their ranks as per the network analysis based on connectivity, perturbation and disruption scores.

Protein	Rank				
	Hub analysis	Perturbation analysis	Disruption analysis	Average	Cumulative
GAPDH	1	1	1	1	1
HSPA8	2	2	3	2.33	2
HSP90AA1	3	3	13	6.33	3
HSPA4	4	6	9	6.33	3
ENO1	6	4	16	8.67	4
TPI1	7	19	2	9.33	5
VCP	11	12	5	9.33	5
CCT2	5	10	15	10	6
HSPA5	12	13	6	10.33	7
LCN2	6	18	8	10.67	8
EEF2	18	8	7	11	9
ACTG1	15	9	10	11.33	10
HSPD1	8	16	12	12	11
PDIA6	16	7	14	12.33	12
CCT8	9	11	17	12.33	12
S100A7	17	20	4	13.67	13
HSPA9	13	17	11	13.67	13
LCN1	19	5	19	14.33	14
HSP90AB1	10	15	20	15	15
CCT5	14	14	18	15.33	16

**Table 2:** Shortlisted proteins from the network analysis of rfhSP-D interactome with reported expression in prostate, prostate cancer, prostate cancer metastasis and their association with apoptosis.

Protein	Expression/Association*			
	Prostate expression^a^	Prostate cancer^b^	Prostate cancer metastasis^b^	Apoptosis^c^
GAPDH	✓	✓	x	✓
HSPA8	✓	✓	x	✓
HSP90AA1	x	✓	✓	✓
HSPA4	✓	✓	x	✓
TPI1	✓	x	x	x
ENO1	✓	✓	x	✓
VCP	✓	✓	x	✓
CCT2	✓	✓	x	✓
HSPA5	✓	✓	✓	✓
LCN2	x	✓	x	✓
EEF2	✓	✓	x	✓
ACTG1	✓	✓	x	x
HSPD1	✓	✓	x	✓
PDIA6	✓	x	x	x
CCT8	✓	x	x	x
S100A7	x	✓	x	✓
HSPA9	✓	✓	x	✓
LCN1	x	x	x	x
HSP90AB1	✓	✓	x	✓
CCT5	✓	x	x	✓

^a^As per data available in protein atlas; ^b^as per data available in DisGeNET; ^C^as per data available in ApocanD and IPA. *Association with apoptosis pathway.

### *In Silico* Validation of the Interaction of SP-D With GRP78

**Figure 6:**
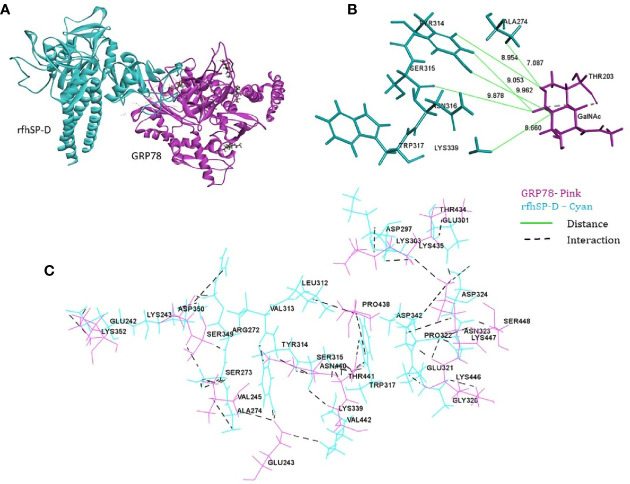
*In silico* validation of the interaction of rfhSP-DSP-D with GRP78. **(A)** Top-ranked docked pose of rfhSP-D (CRD) depicted in cyan and GRP78 (SBD) depicted in pink; **(B)** N-acetyl galactosamine (GalNAc) modeled at Thr203 of GRP78 (pink) was found to be within 10 Å radius of residues of rfhSP-D (cyan) in the docked complex; **(C)** residues involved in intermolecular interactions of GRP78 (pink) and rfhSP-D (cyan).

From the proteomic analysis, GRP78 was the top-ranked protein of the rfhSP-D-PC3 membrane interactome and hypothesized to be involved in the apoptosis signaling by binding to rfhSP-D molecule. *In silico* molecular docking was performed to validate this hypothesis. The top ranked pose (Zrank = −131.3) from blind docking of rfhSP-D and GRP78 revealed that CRD of SP-D can bind with GRP78 *via* the substrate binding domain of GRP78 (**Figure 6**; **Table 3**).

**Table 3:** Intermolecular interactions of GRP78 and rfhSP-D in the top ranked docked pose.

Interactions		Residues	
		GRP78	rfhSP-D
Hydrogen bond	Conventional hydrogen bond	Thr434	Glu301
		Asn440	Val313
		Asn440	Ser315
		Thr441	Trp317
		Val442	Ser315
		Lys446	Glu321
		Ser448	Pro322
	Carbon hydrogen bond	Glu243	Ala274
		Ser349	Arg272
		Ser349	Ser273
		Asp350	Lys243
		Lys435	Lys303
		Asn440	Tyr314
		Lys446	Gly320
		Lys446	Pro322
		Lys447	Pro322
		Ser448	Asn323
Electrostatic		Asp350	Arg272
		Lys352	Glu242
		Lys447	Asp324
		Lys447	Asp342
Salt bridge		Glu243	Lys339
		Lys447	Asp297
Hydrophobic		Val245	Ala274
		Lys435	Lys303
		Pro438	Leu312
		Pro438	Trp317
		Lys447	Pro322

### *In Vitro* Verification of Interaction Between rfhSP-D/rFLhSP-D and GRP78

**Figure 7:**
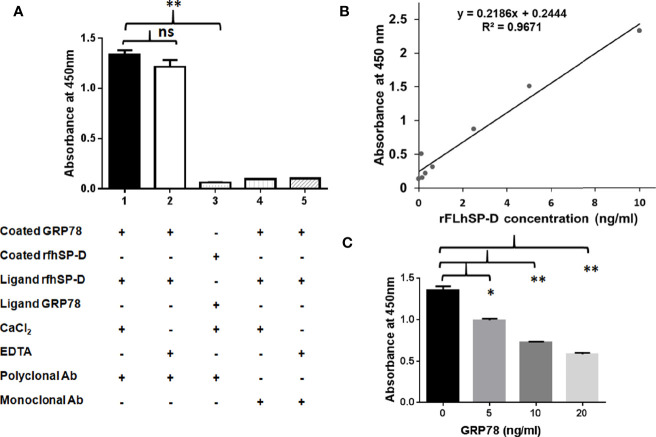
Recombinant human GRP78 binds CRD domain of rfhSP-D/rFLhSP-D. **(A)** Recombinant human GRP78 showed direct binding to rfhSP-D in presence of CaCl_2_ (Bar 1) and was not inhibited by (Bar 2). Monoclonal antibodies against the CRD domain of rfhSP-D did not recognize rfhSP-D bound to recombinant human GRP78 (Bar 3). Recombinant human GRP78 did not interact with rfhSP-D bound to monoclonal antibodies specific to the CRD domain of SP-D (Bar 4). The data presented are the mean ± S.D from independent experiments conducted three times. *p < 0.05 *vs.* Bar 1. **(B)** Standard curve of the rFLhSP-D (0–10 ng/ml) from Duoset SP-D ELISA kit, R & D Systems. **(C)** The rFLhSP-D binding to anti-human SP-D capture antibody (Duoset) was significantly inhibited in the presence of GRP78 in a dose-dependent manner. The GRP78 concentration ‘0’ in the graph represents the control where only buffer was coated and the experiments were conducted three times, *p < 0.05 *vs.* control. **p<0.01 vs. control, ns is non significant.

To examine GRP78 binding to rfhSP-D, we carried out a direct ELISA with recombinant GRP78 and rfhSP-D that was probed using the polyclonal and monoclonal antibodies to SP-D (**Figure 7A**). Polyclonal antibodies could detect rfhSP-D (1 µg/ml, 0.232 nM) bound to coated GRP78 (1 µg/ml, 0.280 nM) in the presence of Ca^2+^. However, EDTA did not significantly inhibit the binding, indicating a protein–protein interaction. Monoclonal anti-human SP-D antibodies recognizing peptides NEAAFLSMTDSK (positions 308–319) and SAAENAALQQLVVAK (positions 293–307) that are located in the CRD region of SP-D (45), could not detect the rfhSP-D bound to coated GRP78, suggesting involvement of the CRD region of rfhSP-D in binding to GRP78.

To evaluate if GRP78 binding can interfere with the interaction of rfhSP-D and polyclonal antibodies, a direct ELISA was carried out using rfhSP-D coated on the microtitre wells, followed by GRP78 as the ligand and probed with polyclonal antibodies. Minimal binding of polyclonal anti-human SP-D antibody to rfhSP-D (1 µg/ml *i.e.* 0.232 nM) was observed due to the blocking/masking of antibody-recognized domains by GRP78 (1 µg/ml *i.e.* 0.280 nM).

To examine if GRP78 binding to rFLhSP-D (recombinant full-length human SP-D) is *via* CRD domain, we carried out a competitive sandwich ELISA (R&D ELISA kit) using the monoclonal anti-human SP-D antibody. The assay detected rFLhSP-D from 0.115 to 10 ng/ml in a linear standard curve (**Figure 7B**). rFLhSP-D bound GRP78 in a dose-dependent manner leading to dose dependent inhibition of binding of monoclonal anti-SP-D detection antibody (**Figure 7C**). GRP78 (20 ng/ml *i.e.* 281 × 10^−3^ nM) on binding to rfhSP-D (5 ng/ml *i.e.* 116 × 10^−3^ nM) showed approximately 57.15% inhibition of binding to monoclonal anti-human SP-D antibodies. Thus, GRP78 interfered with the interaction between rFLhSP-D and monoclonal anti-human SP-D antibody, suggesting that CRD of rFLhSP-D is likely to be involved in its binding to GRP78.

## Discussion

To delineate the underlying mechanisms of the anti-prostate cancer activity of rfhSP-D, we used a pull-down strategy to unravel the rfhSP-D interactome from the membrane fraction of the metastatic prostate cancer cell line, PC3. A high-throughput proteomic work flow led to the identification of 347 membrane proteins with ≥2 unique peptides, each with ≥2 PSMs. Based on the PPi network properties such as connectivity, perturbation, and disruption scores, 20 proteins were selected, including GRP78 that probably interacted directly or indirectly with rfhSP-D. The interaction between rfhSP-D and GRP78 was validated by *in silico* and *in vitro* experimental analysis.

We recently reported a differentially increased binding of rfhSP-D to the metastatic prostate cancer cells and induction of the apoptosis (16). The membrane interactome of PC3 cells, treated with rfhSP-D, showed involvement of several heat-shock protein family members such as GRP78 (HSPA5), HSP90AB1, HSPA8, HSP90AA1, HSPA1B, HSP90B1, and HSPD1, as previously reported (46). Elevated levels of HSPs have been reported in many cancers, including prostate cancer. Both GRP78 and HSP90AA1 were present in the databases of prostate cancer (C0376358), metastasis of prostate cancer (C1282496) using data available in DisGeNET, and have a significant role in cancer cell apoptosis and survival as per information in ApocanD database and IPA. However, HSP90AA1 is not expressed in the prostate glandular cells as per protein atlas data (26). In addition, it does not relocate to the cell membrane like GRP78 in prostate cancer and is rather secreted extracellularly (47). Interestingly, the ‘Cellular and Molecular Pathway’ analysis of rfhSP-D interactome listed GRP78 and SP-D together in the top ranked ‘Cell Death and Survival’ category. Hsp90AB1 induces angiogenesis in the hepatocellular carcinomas, promotes EMT in gastric cancer, and its upregulated expression is implicated in metastasis and differentiation of lung cancer (48–50). HSPA1B and HSPA8 have shown involvement in the apoptotic signaling. Proteomics analysis of endometrial carcinoma tissue identified HSPA8 as the most upregulated candidate, and siRNA-mediated inhibition of HSPA8 significantly downregulated cell proliferation and promoted cell apoptosis in RL-95-2 and HEC-1B, two endometrial cancer cell lines (51). siRNA targeting HSPA1B gene inhibited proliferation of HeLa, MCF-7, PC-3, HuH-7, and gastric cancer SGC-7901 cells, while the non-tumorigenic HBL-100 mammary cells were not affected adversely (52). HSP90B1, a stress-inducible chaperone protein, significantly reduced the cell proliferation and survival of the malignant cells (53). In comparison with the benign prostate samples, HSPD1/E1 Complex is over-expressed in prostate cancer lesions and in carcinomas (54).

GRP78, also called heat-shock protein 5 (HSPA5), is a member of the HSP70 superfamily, and has a critical role in the regulation of the unfolded protein response (UPR) *via* appropriate protein folding, inhibiting aggregation of newly-made proteins, and regulating the stimulation of transmembrane sensors in the endoplasmic reticulum (ER) (55). Expression of GRP78 is significantly enhanced in various cancers, and is linked with the prostate cancer malignancy, metastasis and acquisition of resistance to chemotherapy (56). Prostate tumorigenesis was potently arrested in bi-allelic conditional knockout mice for both GRP78 and PTEN (57). Environment of the fast-growing solid tumors is marked with increased hypoxia, and reduced nutrients and acidosis, leading to UPR and increased GRP78 expression. GRP78 directly interacted with apoptotic pathway intermediates to block caspase activation, and eventually led to increased cell survival (58–60).

GRP78 has been primarily localized to the ER owing to the presence of the KDEL-retention motif, although a minor proportion of GRP78 evades the ER-retention mechanism and reaches the cell surface to promote cell survival (61). Proteomics of the membranes of various tumor cells unravelled a number of heat-shock chaperones and glucose-regulated proteins, including GRP78 (62). These reports are in coherence with our observation of GRP78 to be in the membrane proteome of PC3 cells. Additionally, Thapsigargin, a mediator of ER stress, is involved in surface localization of GRP78 in 293T, HeLa, and MCF-7 cell lines (63). Furthermore, an increased GRP78 expression can lead to its translocation to the membrane even when there is no ER stress (64).

Membrane GRP78 may bind to *α*2-macroglobulin (*α*2-M), tumor differentiation factor, and vaspin *via* its substrate binding domain and induce AKT/PI3K pro-survival pathway (65–71). Peptides targeting membrane GRP78 induce selective tumor cell death. Antibody ligation to cell-surface GRP78 slowed growth rate in prostate cancer cells and blocked PI3K/AKT signaling (72). The predicted involvement of the substrate binding domain of GRP78 in the interaction with rfhSP-D suggested that SP-D may interfere with these pro-survival mechanisms. Thus, the rfhSP-D induced inhibition of PI3K/Akt pathway leading to apoptosis of prostate cancer cells could be plausibly mediated by GRP78 (16).

Case–control studies of lung cancer patients have revealed that the circulating SP-D levels may predict susceptibility to lung cancer (73, 74). The collagen and CRD regions of SP-D play different but vital roles in the immune surveillance. The CRD region mediates the pattern recognition function, while the collagen region remains important for the signaling interaction *via* Calreticulin–CD91 complex. However, studies using rfhSP-D have revealed that the homotrimeric neck and CRD region are endowed with virtual self-sufficiency in many aspects (6). Several lines of evidence suggest the importance of CRD region of SP-D in its biological activity, *e.g.* CRD region interfered with the EGF and EGF receptor (EGFR) interaction, causing downregulation of the EGF induced signaling in A549 cell line (11). EGF–EGFR interaction results in increased epithelial tumor cell proliferation, angiogenic differentiation, and invasive capability, leading to increased probability of metastasis. Recently, it was demonstrated that SP-D interacted with the EGFR mutant and interfered with its dimerization that was independent of its interaction with EGF (75). The interaction of SP-D and rfhSP-D with eosinophilic leukemic AML cells was also mediated by CRD (9, 10). Kaur et al. proposed that the CRD region of SP-D is involved in binding to target ligand on the pancreatic cancer cell surface *via* protein–protein interaction (13). Our results demonstrate that the interaction of rfhSP-D with GRP78 also involved the CRD domain, which could be relevant in the induction of apoptosis in cancer cells.

O-glycosylation of GRP78 is critical for its anti-apoptotic function (76). Blind docking of the crystal structures of active rfhSP-D and monomeric glycosylated structure of GRP78 revealed that the CRD of rfhSP-D can bind GRP78 *via* the substrate binding domain of GRP78. Thus, our *in silico* and *in vitro* analyses validate the interaction between rFLhSP-D/rfhSP-D and GRP78 *via* CRD region, substantiating GRP78 as binding partner of SP-D and may potentially be a novel intermediary in SP-D mediated innate immune surveillance against prostate cancer. The therapeutic relevance of SP-D-GRP78 interaction may be further explored using *in vivo* studies with knockout mice models bearing prostate cancer.

## Data Availability Statement

The datasets presented in this study can be found in online repositories. The names of the repositories and accession number(s) can be found in the article/**Supplementary Material**.

## Author Contributions

Conception, co-ordination, and design of the study were accomplished by GT and TM. GT performed and analyzed the experiments and prepared the first draft of the manuscript. SA and UK cloned, recombinantly expressed, and characterized the purified rfhSP-D used in the study. RS, PG, and GS designed, performed, and analyzed the LC-MS/MS data. SI-T and IK designed, executed, and analyzed network and *in silico* molecular docking studies. PG and BB performed and analyzed LC-MS/MS data by IPA software. SI-T, BB, PG, RS, and UK reviewed the first draft and provided valuable suggestions. TM defended the proposal to acquire the Institutional grant and IEC approval, analyzed the data, and edited the manuscript. All authors contributed to the article and approved the submitted version.

## Funding

Intra-mural grant allocated to TM by the Institute ICMR-NIRRH (Accession no. 921) was used for all the consumables used for experiments. ICMR-NIRRH-JRF and ICMR-SRF supported GT’s salary.

## Conflict of Interest

The authors declare that the research was conducted in the absence of any commercial or financial relationships that could be construed as a potential conflict of interest.

## Supplementary Material

Supplementary Figure 1 Graphical abstract of the study.

Supplementary File S2 List of 347 proteins from LC-MS/MS analysis with their accession, gene symbol, description, protein name, molecule weight, sequence coverage (%), PSMs, unique peptides, and Score Sequest HT: Sequest HT.

Supplementary File S3 The PPI network of 347 proteins was analyzed for network parameters to shortlist critical proteins of the rfhSP-D interactome. 20 proteins were selected and ranked based on connectivity, perturbation, and disruption scores.
