# Supplementary figures and images for: Membrane Interactome of a Recombinant Fragment of Human Surfactant Protein D Reveals GRP78 as a Novel Binding Partner in PC3, a Metastatic Prostate Cancer Cell Line
Source: Front Immunol. 2021 Jan 19;11:600660. doi: 10.3389/fimmu.2020.600660 (PMC7850985; doi:10.3389/fimmu.2020.600660)

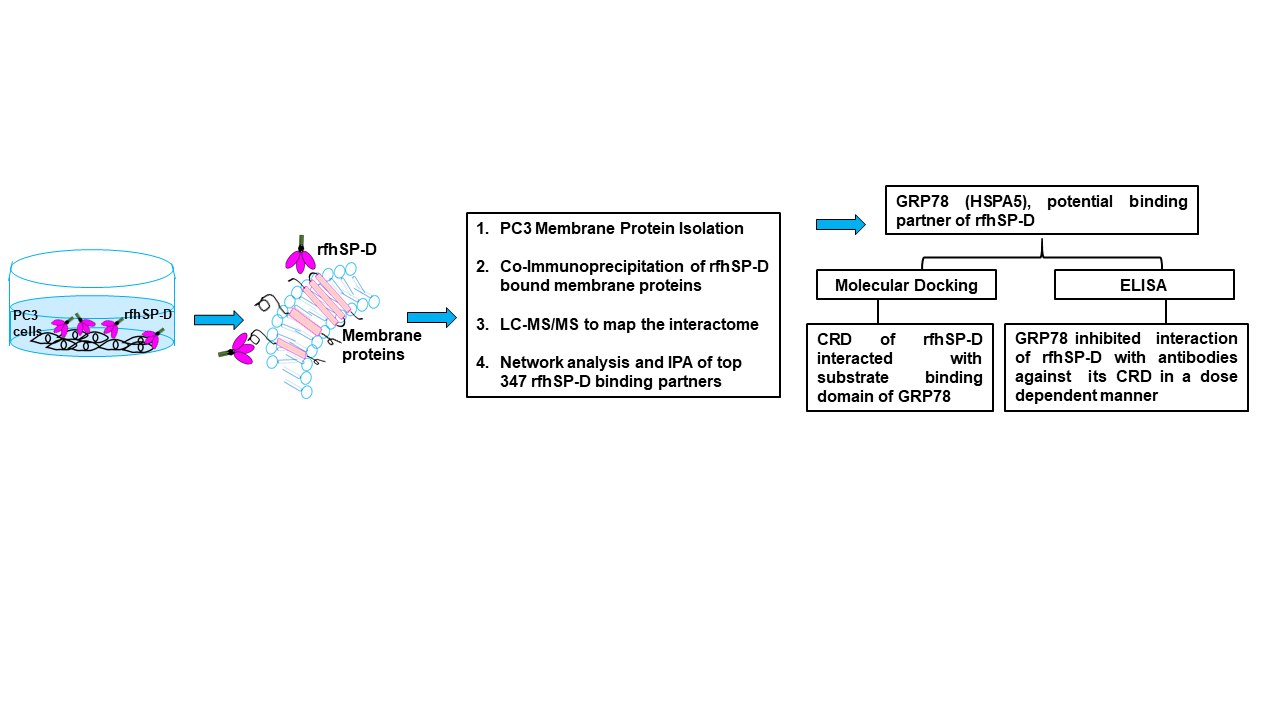

Supplement: Supplementary Figure 1 — Graphical abstract of the study. [file Image_1.tif]
