# Supplementary material for: Membrane Interactome of a Recombinant Fragment of Human Surfactant Protein D Reveals GRP78 as a Novel Binding Partner in PC3, a Metastatic Prostate Cancer Cell Line
Source: Front Immunol. 2021 Jan 19;11:600660. doi: 10.3389/fimmu.2020.600660 (PMC7850985; doi:10.3389/fimmu.2020.600660)
Supplement: Supplementary file 2 [file Table_1.docx]

**Supplementary Table 1. List of 347 proteins from LC-MS/MS analysis detected with**

**925 ≥2 unique peptides, each with at least 2 peptide-spectrum matches (PSMs).**

Seven members of HSP family (including GRP78 or HSAP5) were in the top fifteen identified proteins. Four previously known SP-D receptors [DEFA1 (Defensin), CALR (calreticulin), C1QBP (C1q receptor, gC1qR), and A2ML1 (alpha-2-macroglobulin-like protein 1] have been identified in the list.

| **Gene Symbol** | **Description** | **Peptides** | **PSMs** | **MW [kDa]** |
| --- | --- | --- | --- | --- |
| HSPA5 | 78 kDa glucose-regulated protein precursor | 19 | 72 | 72.3 |
| ACTN1 | PREDICTED: alpha-actinin-1 isoform X4 | 26 | 70 | 121.9 |
| ACTN4 | PREDICTED: alpha-actinin-4 isoform X3 | 22 | 61 | 107.2 |
| HSP90AB1 | heat shock protein HSP 90-beta isoform a | 16 | 55 | 83.2 |
| HSPA8 | PREDICTED: heat shock cognate 71 kDa protein isoform X1 | 16 | 54 | 70.9 |
| HSP90AA1 | heat shock protein HSP 90-alpha isoform 1 | 15 | 54 | 98.1 |
| ANXA2 | annexin A2 isoform 1 | 14 | 50 | 40.4 |
| ENO1 | alpha-enolase isoform 1 | 14 | 46 | 47.1 |
| P4HB | protein disulfide-isomerase precursor | 12 | 42 | 57.1 |
| HSPA1B | heat shock 70 kDa protein 1B | 11 | 39 | 70 |
| AHNAK | PREDICTED: neuroblast differentiation-associated protein AHNAK isoform X2 | 19 | 37 | 621.4 |
| HSP90B1 | endoplasmin precursor | 14 | 36 | 92.4 |
| HSPD1 | 60 kDa heat shock protein, mitochondrial | 13 | 35 | 61 |
| ANXA6 | annexin A6 isoform 1 | 11 | 32 | 75.8 |
| PIGR | PREDICTED: polymeric immunoglobulin receptor isoform X1 | 11 | 31 | 83.2 |
| ANXA1 | PREDICTED: annexin A1 isoform X1 | 11 | 31 | 40.1 |
| KRT1 | keratin, type II cytoskeletal 1 | 13 | 31 | 66 |
| KRT10 | PREDICTED: keratin, type I cytoskeletal 10 isoform X1 | 12 | 31 | 63.3 |
| VCL | vinculin isoform meta-VCL | 15 | 29 | 123.7 |
| KRT9 | keratin, type I cytoskeletal 9 | 12 | 29 | 62 |
| ATP5B | ATP synthase subunit beta, mitochondrial precursor | 9 | 29 | 56.5 |
| PGK1 | phosphoglycerate kinase 1 | 7 | 29 | 44.6 |
| FLNB | filamin-B isoform 1 | 15 | 28 | 281.5 |
| VIM | Vimentin | 10 | 28 | 53.6 |
| TPI1 | triosephosphate isomerase isoform 2 | 8 | 28 | 30.8 |
| S100A7 | protein S100-A7 | 5 | 28 | 11.5 |
| MUC5B | mucin-5B precursor | 10 | 27 | 596 |
| HSPA9 | stress-70 protein, mitochondrial precursor | 11 | 26 | 73.6 |
| CALR | calreticulin precursor | 10 | 25 | 48.1 |
| PIP | prolactin-inducible protein precursor | 6 | 25 | 16.6 |
| PLS3 | plastin-3 isoform 1 | 11 | 25 | 70.8 |
| ACTG1 | actin, cytoplasmic 2 | 6 | 24 | 41.8 |
| ANXA11 | PREDICTED: annexin A11 isoform X1 | 7 | 23 | 65.6 |
| HNRNPK | PREDICTED: heterogeneous nuclear ribonucleoprotein K isoform X3 | 7 | 22 | 48.5 |
| TAGLN2 | transgelin-2 isoform a | 8 | 21 | 24.4 |
| CCT3 | T-complex protein 1 subunit gamma isoform a | 8 | 21 | 60.5 |
| KRT13 | keratin, type I cytoskeletal 13 isoform a | 9 | 21 | 49.5 |
| PDIA4 | PREDICTED: protein disulfide-isomerase A4 isoform X1 | 7 | 20 | 73 |
| PDIA3 | protein disulfide-isomerase A3 precursor | 6 | 20 | 56.7 |
| PRDX1 | peroxiredoxin-1 | 6 | 20 | 22.1 |
| PSAP | prosaposin isoform b preproprotein | 7 | 19 | 58.4 |
| HBB | hemoglobin subunit beta | 6 | 19 | 16 |
| CFL1 | cofilin-1 | 6 | 19 | 18.5 |
| PPA1 | inorganic pyrophosphatase | 6 | 19 | 32.6 |
| PKM | pyruvate kinase PKM isoform c | 9 | 18 | 65.9 |
| CALU | calumenin isoform c precursor | 6 | 18 | 38 |
| PFN1 | profilin-1 | 6 | 18 | 15 |
| ATP5A1 | PREDICTED: ATP synthase subunit alpha, mitochondrial isoform X1 | 6 | 18 | 64.1 |
| COL6A1 | collagen alpha-1(VI) chain precursor | 5 | 18 | 108.5 |
| YWHAZ | PREDICTED: 14-3-3 protein zeta/delta isoform X1 | 6 | 18 | 27.7 |
| LCP1 | plastin-2 | 9 | 18 | 70.2 |
| APOA1 | apolipoprotein A-I isoform 1 preproprotein | 7 | 17 | 30.8 |
| PRDX6 | peroxiredoxin-6 | 7 | 17 | 25 |
| MDH2 | malate dehydrogenase, mitochondrial isoform 1 precursor | 6 | 17 | 35.5 |
| KRT19 | keratin, type I cytoskeletal 19 | 8 | 17 | 44.1 |
| PEBP1 | phosphatidylethanolamine-binding protein 1 | 5 | 17 | 21 |
| CLU | PREDICTED: clusterin isoform X1 | 5 | 17 | 57.8 |
| PRKCSH | glucosidase 2 subunit beta isoform 3 precursor | 7 | 16 | 60.2 |
| TFRC | transferrin receptor protein 1 isoform 1 | 7 | 16 | 84.8 |
| SERPINA1 | alpha-1-antitrypsin precursor | 6 | 16 | 46.7 |
| PGAM1 | phosphoglycerate mutase 1 isoform 1 | 6 | 16 | 28.8 |
| CCT8 | T-complex protein 1 subunit theta isoform 1 | 6 | 16 | 59.6 |
| CALML5 | calmodulin-like protein 5 | 5 | 16 | 15.9 |
| NME1 | nucleoside diphosphate kinase A isoform a | 5 | 16 | 19.6 |
| GAPDH | glyceraldehyde-3-phosphate dehydrogenase isoform 1 | 4 | 16 | 36 |
| S100A9 | protein S100-A9 | 3 | 16 | 13.2 |
| ANXA5 | annexin A5 | 6 | 15 | 35.9 |
| PABPC1 | polyadenylate-binding protein 1 | 6 | 15 | 70.6 |
| HNRNPA1 | heterogeneous nuclear ribonucleoprotein A1 isoform b | 6 | 15 | 38.7 |
| PRDX5 | peroxiredoxin-5, mitochondrial isoform a precursor | 5 | 15 | 22 |
| RHOC | rho-related GTP-binding protein RhoC precursor | 4 | 15 | 22 |
| HSPH1 | heat shock protein 105 kDa isoform 1 | 8 | 14 | 96.8 |
| STIP1 | stress-induced-phosphoprotein 1 isoform a | 6 | 14 | 68 |
| CLIC1 | chloride intracellular channel protein 1 | 6 | 14 | 26.9 |
| EEF2 | elongation factor 2 | 6 | 14 | 95.3 |
| PDIA6 | protein disulfide-isomerase A6 isoform a | 5 | 14 | 53.9 |
| UBA1 | PREDICTED: ubiquitin-like modifier-activating enzyme 1 isoform X1 | 5 | 14 | 123.1 |
| ALDOA | fructose-bisphosphate aldolase A isoform 2 | 6 | 14 | 45.2 |
| APEH | acylamino-acid-releasing enzyme | 6 | 13 | 81.2 |
| ITGB1 | integrin beta-1 isoform 1A precursor | 5 | 13 | 88.4 |
| LRPPRC | leucine-rich PPR motif-containing protein, mitochondrial precursor | 5 | 13 | 157.8 |
| TPD52L2 | tumor protein D54 isoform a | 5 | 13 | 24.8 |
| HNRNPA2B1 | PREDICTED: heterogeneous nuclear ribonucleoproteins A2/B1 isoform X1 | 5 | 13 | 37.4 |
| EEF1D | PREDICTED: elongation factor 1-delta isoform X1 | 5 | 13 | 75.6 |
| HSPB1 | heat shock protein beta-1 | 4 | 13 | 22.8 |
| TUBA1B | tubulin alpha-1B chain | 4 | 13 | 50.1 |
| GPI | PREDICTED: glucose-6-phosphate isomerase isoform X1 | 4 | 13 | 67.2 |
| UBA52 | PREDICTED: ubiquitin-60S ribosomal protein L40 isoform X1 | 3 | 13 | 22.2 |
| FLNA | filamin-A isoform 2 | 6 | 12 | 280.6 |
| GM2A | ganglioside GM2 activator isoform 1 precursor | 5 | 12 | 20.8 |
| LDHB | L-lactate dehydrogenase B chain isoform LDHBx | 5 | 12 | 37.4 |
| CCT4 | T-complex protein 1 subunit delta isoform a | 5 | 12 | 57.9 |
| HNRNPC | PREDICTED: heterogeneous nuclear ribonucleoproteins C1/C2 isoform X1 | 5 | 12 | 33.7 |
| NPM1 | nucleophosmin isoform 1 | 4 | 12 | 32.6 |
| NSFL1C | PREDICTED: NSFL1 cofactor p47 isoform X1 | 4 | 12 | 41.2 |
| SOD1 | superoxide dismutase [Cu-Zn] | 3 | 12 | 15.9 |
| YWHAE | 14-3-3 protein epsilon | 4 | 12 | 29.2 |
| CSTB | cystatin-B | 2 | 12 | 11.1 |
| KRT6B | keratin, type II cytoskeletal 6B | 5 | 12 | 60 |
| PSME1 | proteasome activator complex subunit 1 isoform 2 | 5 | 11 | 28.6 |
| FAM49B | PREDICTED: protein FAM49B isoform X1 | 5 | 11 | 36.7 |
| AZGP1 | zinc-alpha-2-glycoprotein precursor | 4 | 11 | 34.2 |
| C1QBP | complement component 1 Q subcomponent-binding protein, mitochondrial precursor | 4 | 11 | 31.3 |
| HPCAL1 | hippocalcin-like protein 1 | 4 | 11 | 22.3 |
| FABP5 | fatty acid-binding protein, epidermal | 4 | 11 | 15.2 |
| GOT2 | aspartate aminotransferase, mitochondrial isoform 1 precursor | 4 | 11 | 47.5 |
| ANXA7 | annexin A7 isoform 2 | 4 | 11 | 52.7 |
| DDX39B | spliceosome RNA helicase DDX39B | 4 | 11 | 49 |
| TTR | transthyretin precursor | 3 | 11 | 15.9 |
| PCBP1 | poly(rC)-binding protein 1 | 4 | 11 | 37.5 |
| PDLIM1 | PDZ and LIM domain protein 1 | 3 | 11 | 36 |
| CANX | PREDICTED: calnexin isoform X2 | 3 | 11 | 73.4 |
| KHSRP | PREDICTED: far upstream element-binding protein 2 isoform X3 | 4 | 11 | 70.4 |
| YWHAB | PREDICTED: 14-3-3 protein beta/alpha isoform X1 | 3 | 11 | 28.1 |
| CTSD | cathepsin D preproprotein | 5 | 10 | 44.5 |
| LCN1 | PREDICTED: lipocalin-1 isoform X1 | 5 | 10 | 31.6 |
| NCL | Nucleolin | 4 | 10 | 76.6 |
| TWF2 | twinfilin-2 | 4 | 10 | 39.5 |
| CAPZA1 | PREDICTED: F-actin-capping protein subunit alpha-1 isoform X1 | 4 | 10 | 36.5 |
| FKBP3 | PREDICTED: peptidyl-prolyl cis-trans isomerase FKBP3 isoform X1 | 4 | 10 | 26 |
| RPL12 | 60S ribosomal protein L12 | 3 | 10 | 17.8 |
| IGLL5 | immunoglobulin lambda-like polypeptide 5 isoform 1 | 3 | 10 | 23 |
| KRT8 | keratin, type II cytoskeletal 8 isoform 1 | 3 | 10 | 56.6 |
| CLTC | PREDICTED: clathrin heavy chain 1 isoform X1 | 6 | 9 | 192.3 |
| DLD | dihydrolipoyl dehydrogenase, mitochondrial isoform 1 precursor | 4 | 9 | 54.1 |
| CSE1L | exportin-2 isoform 1 | 4 | 9 | 110.3 |
| EEF1G | elongation factor 1-gamma | 4 | 9 | 50.1 |
| ELAVL1 | ELAV-like protein 1 | 4 | 9 | 36.1 |
| PCBP2 | poly(rC)-binding protein 2 isoform a | 5 | 9 | 38.6 |
| PRDX3 | thioredoxin-dependent peroxide reductase, mitochondrial isoform a precursor | 4 | 9 | 27.7 |
| CASP14 | caspase-14 precursor | 4 | 9 | 27.7 |
| COL1A1 | collagen alpha-1(I) chain preproprotein | 3 | 9 | 138.8 |
| ALDH1A3 | aldehyde dehydrogenase family 1 member A3 isoform 1 | 3 | 9 | 56.1 |
| TXNRD1 | thioredoxin reductase 1, cytoplasmic isoform 3 | 3 | 9 | 70.9 |
| ACO2 | aconitate hydratase, mitochondrial precursor | 3 | 9 | 85.4 |
| DBI | acyl-CoA-binding protein isoform 4 | 3 | 9 | 16.5 |
| KRT7 | keratin, type II cytoskeletal 7 | 4 | 9 | 51.4 |
| TALDO1 | Transaldolase | 3 | 9 | 37.5 |
| ERO1A | ERO1-like protein alpha precursor | 3 | 9 | 54.4 |
| CYB5R3 | NADH-cytochrome b5 reductase 3 isoform 3 | 2 | 9 | 38.2 |
| RCN1 | reticulocalbin-1 precursor | 2 | 9 | 38.9 |
| TXNDC17 | thioredoxin domain-containing protein 17 | 2 | 9 | 13.9 |
| LDHA | L-lactate dehydrogenase A chain isoform 3 | 5 | 8 | 39.8 |
| HSPA4 | heat shock 70 kDa protein 4 | 6 | 8 | 94.3 |
| PSMD2 | 26S proteasome non-ATPase regulatory subunit 2 isoform 1 | 5 | 8 | 100.1 |
| C3 | complement C3 preproprotein | 4 | 8 | 187 |
| PYGB | glycogen phosphorylase, brain form | 5 | 8 | 96.6 |
| HSPA13 | heat shock 70 kDa protein 13 precursor | 4 | 8 | 51.9 |
| MYL12A | myosin regulatory light chain 12A isoform 2 | 3 | 8 | 20.4 |
| KLK3 | prostate-specific antigen isoform 1 preproprotein | 3 | 8 | 28.7 |
| HSPE1 | 10 kDa heat shock protein, mitochondrial | 3 | 8 | 10.9 |
| LGALS1 | galectin-1 | 3 | 8 | 14.7 |
| CSTA | cystatin-A | 3 | 8 | 11 |
| CBX1 | chromobox protein homolog 1 | 3 | 8 | 21.4 |
| MYL6 | myosin light polypeptide 6 isoform 1 | 3 | 8 | 16.9 |
| PAEP | PREDICTED: glycodelin isoform X1 | 3 | 8 | 23 |
| HDGF | hepatoma-derived growth factor isoform d | 2 | 8 | 29 |
| S100A8 | protein S100-A8 isoform a | 2 | 8 | 13.5 |
| FKBP4 | peptidyl-prolyl cis-trans isomerase FKBP4 | 2 | 8 | 51.8 |
| ST13 | hsc70-interacting protein isoform 1 | 2 | 8 | 41.3 |
| PRDX2 | peroxiredoxin-2 | 3 | 8 | 21.9 |
| CYCS | cytochrome c | 2 | 8 | 11.7 |
| HNRNPAB | heterogeneous nuclear ribonucleoprotein A/B isoform a | 3 | 8 | 35.9 |
| NACA | PREDICTED: nascent polypeptide-associated complex subunit alpha isoform X1 | 2 | 8 | 205.6 |
| SERPINB13 | serpin B13 isoform 1 | 4 | 7 | 45.3 |
| HBA1 | hemoglobin subunit alpha | 3 | 7 | 15.2 |
| VDAC2 | voltage-dependent anion-selective channel protein 2 isoform 1 | 3 | 7 | 33.4 |
| PSMA7 | proteasome subunit alpha type-7 | 3 | 7 | 27.9 |
| FSCN1 | Fascin | 3 | 7 | 54.5 |
| RAB11B | ras-related protein Rab-11B | 3 | 7 | 24.5 |
| EIF1B | eukaryotic translation initiation factor 1b | 3 | 7 | 12.8 |
| GLO1 | lactoylglutathione lyase | 3 | 7 | 20.8 |
| EIF4H | PREDICTED: eukaryotic translation initiation factor 4H isoform X1 | 3 | 7 | 27.6 |
| DEFA1 | PREDICTED: neutrophil defensin 1 isoform X1 | 3 | 7 | 11 |
| TF | PREDICTED: serotransferrin isoform X1 | 3 | 7 | 77 |
| KRT5 | keratin, type II cytoskeletal 5 | 4 | 7 | 62.3 |
| RPLP2 | 60S acidic ribosomal protein P2 | 2 | 7 | 11.7 |
| CLTA | clathrin light chain A isoform a | 2 | 7 | 23.6 |
| YWHAQ | 14-3-3 protein theta | 3 | 7 | 27.7 |
| RBMX | RNA-binding motif protein, X chromosome isoform 1 | 4 | 6 | 42.3 |
| AP2B1 | PREDICTED: AP-2 complex subunit beta isoform X1 | 4 | 6 | 109.3 |
| FH | fumarate hydratase, mitochondrial | 3 | 6 | 54.6 |
| PPIB | peptidyl-prolyl cis-trans isomerase B precursor | 3 | 6 | 23.7 |
| PDCD6IP | programmed cell death 6-interacting protein isoform 2 | 3 | 6 | 96.7 |
| GC | vitamin D-binding protein isoform 3 precursor | 3 | 6 | 55 |
| TNPO1 | transportin-1 isoform 1 | 3 | 6 | 102.3 |
| SSB | lupus La protein | 3 | 6 | 46.8 |
| TGM3 | protein-glutamine gamma-glutamyltransferase E | 3 | 6 | 76.6 |
| UBE2N | ubiquitin-conjugating enzyme E2 N | 3 | 6 | 17.1 |
| FCGBP | IgGFc-binding protein precursor | 3 | 6 | 571.6 |
| ANXA3 | annexin A3 | 3 | 6 | 36.4 |
| S100A11 | protein S100-A11 | 3 | 6 | 11.7 |
| PLIN3 | perilipin-3 isoform 1 | 3 | 6 | 47 |
| PA2G4 | proliferation-associated protein 2G4 | 3 | 6 | 43.8 |
| CNPY2 | protein canopy homolog 2 isoform 1 precursor | 3 | 6 | 20.6 |
| MLEC | malectin isoform1 precursor | 3 | 6 | 32.2 |
| LRRC59 | leucine-rich repeat-containing protein 59 | 3 | 6 | 34.9 |
| FKBP1A | peptidyl-prolyl cis-trans isomerase FKBP1A isoform a | 3 | 6 | 11.9 |
| PTBP1 | PREDICTED: polypyrimidine tract-binding protein 1 isoform X1 | 3 | 6 | 59.8 |
| SET | PREDICTED: protein SET isoform X1 | 3 | 6 | 35.8 |
| TGM1 | protein-glutamine gamma-glutamyltransferase K | 2 | 6 | 89.7 |
| RPS12 | 40S ribosomal protein S12 | 2 | 6 | 14.5 |
| PPP1CA | serine/threonine-protein phosphatase PP1-alpha catalytic subunit isoform 3 | 3 | 6 | 38.6 |
| CD59 | CD59 glycoprotein preproprotein | 2 | 6 | 14.2 |
| DCD | dermcidin isoform 2 preproprotein | 2 | 6 | 12.4 |
| COX6B1 | cytochrome c oxidase subunit 6B1 | 2 | 6 | 10.2 |
| ITGA2 | integrin alpha-2 precursor | 2 | 6 | 129.2 |
| USP14 | ubiquitin carboxyl-terminal hydrolase 14 isoform a | 2 | 6 | 56 |
| PSCA | prostate stem cell antigen preproprotein | 2 | 6 | 12 |
| CBX3 | chromobox protein homolog 3 | 2 | 6 | 20.8 |
| MYDGF | myeloid-derived growth factor precursor | 2 | 6 | 18.8 |
| SH3BGRL3 | SH3 domain-binding glutamic acid-rich-like protein 3 | 2 | 6 | 10.4 |
| HNRNPDL | heterogeneous nuclear ribonucleoprotein D-like isoform a | 3 | 6 | 46.4 |
| SKP1 | S-phase kinase-associated protein 1 isoform b | 2 | 6 | 18.6 |
| KRT18 | keratin, type I cytoskeletal 18 | 4 | 6 | 48 |
| GRN | PREDICTED: granulins isoform X1 | 2 | 6 | 63.5 |
| HLA-A | PREDICTED: HLA class I histocompatibility antigen, A-1 alpha chain isoform X2 | 2 | 6 | 44 |
| JCHAIN | PREDICTED: immunoglobulin J chain isoform X1 | 2 | 6 | 18.1 |
| PTGES3 | PREDICTED: prostaglandin E synthase 3 isoform X1 | 2 | 6 | 19.4 |
| ANPEP | PREDICTED: aminopeptidase N isoform X1 | 5 | 5 | 109.5 |
| SF3A1 | splicing factor 3A subunit 1 | 4 | 5 | 88.8 |
| AHCY | adenosylhomocysteinase isoform 1 | 3 | 5 | 47.7 |
| TUBB | tubulin beta chain isoform a | 3 | 5 | 52 |
| GDI2 | rab GDP dissociation inhibitor beta isoform 1 | 3 | 5 | 50.6 |
| PSMD11 | 26S proteasome non-ATPase regulatory subunit 11 | 3 | 5 | 47.4 |
| ANP32A | acidic leucine-rich nuclear phosphoprotein 32 family member A | 3 | 5 | 28.6 |
| ATP5H | ATP synthase subunit d, mitochondrial isoform a | 3 | 5 | 18.5 |
| COTL1 | coactosin-like protein | 3 | 5 | 15.9 |
| BPIFB1 | BPI fold-containing family B member 1 precursor | 3 | 5 | 52.4 |
| FKBP10 | PREDICTED: peptidyl-prolyl cis-trans isomerase FKBP10 isoform X1 | 3 | 5 | 66.3 |
| CRABP2 | PREDICTED: cellular retinoic acid-binding protein 2 isoform X1 | 3 | 5 | 15.7 |
| HPX | hemopexin precursor | 2 | 5 | 51.6 |
| RANBP1 | ran-specific GTPase-activating protein isoform 1 | 2 | 5 | 31.9 |
| TPD52 | tumor protein D52 isoform 4 | 2 | 5 | 26.9 |
| DYNLRB1 | dynein light chain roadblock-type 1 isoform d | 2 | 5 | 13.4 |
| PCBD1 | pterin-4-alpha-carbinolamine dehydratase isoform 2 | 2 | 5 | 12 |
| DLAT | dihydrolipoyllysine-residue acetyltransferase component of pyruvate dehydrogenase complex, mitochondrial precursor | 2 | 5 | 69 |
| LTF | lactotransferrin isoform 1 preproprotein | 2 | 5 | 78.1 |
| PGC | gastricsin isoform 1 preproprotein | 2 | 5 | 42.4 |
| PSMA6 | proteasome subunit alpha type-6 isoform a | 2 | 5 | 27.4 |
| SCP2 | non-specific lipid-transfer protein isoform 1 precursor | 2 | 5 | 59 |
| UQCRC1 | cytochrome b-c1 complex subunit 1, mitochondrial precursor | 2 | 5 | 52.6 |
| MANF | mesencephalic astrocyte-derived neurotrophic factor precursor | 2 | 5 | 21.1 |
| RTN3 | reticulon-3 isoform a | 2 | 5 | 25.6 |
| CCT2 | T-complex protein 1 subunit beta isoform 1 | 2 | 5 | 57.5 |
| LMAN2 | vesicular integral-membrane protein VIP36 precursor | 2 | 5 | 40.2 |
| SERPINB3 | serpin B3 | 2 | 5 | 44.5 |
| CCT5 | T-complex protein 1 subunit epsilon isoform a | 2 | 5 | 59.6 |
| LETM1 | LETM1 and EF-hand domain-containing protein 1, mitochondrial precursor | 2 | 5 | 83.3 |
| UBQLN1 | ubiquilin-1 isoform 1 | 2 | 5 | 62.5 |
| DNAJC8 | dnaJ homolog subfamily C member 8 | 2 | 5 | 29.8 |
| TXNDC12 | thioredoxin domain-containing protein 12 precursor | 2 | 5 | 19.2 |
| CRNN | Cornulin | 2 | 5 | 53.5 |
| CFL2 | cofilin-2 isoform 1 | 3 | 5 | 18.7 |
| ARF1 | PREDICTED: ADP-ribosylation factor 1 isoform X1 | 2 | 5 | 23.8 |
| SFPQ | PREDICTED: splicing factor, proline- and glutamine-rich isoform X1 | 2 | 5 | 76.1 |
| YBX2 | PREDICTED: Y-box-binding protein 2 isoform X1 | 2 | 5 | 40.2 |
| ITGA3 | integrin alpha-3 preproprotein | 3 | 4 | 116.5 |
| KPNB1 | importin subunit beta-1 isoform 1 | 3 | 4 | 97.1 |
| SUB1 | PREDICTED: activated RNA polymerase II transcriptional coactivator p15 isoform X1 | 3 | 4 | 14.4 |
| ACAT1 | acetyl-CoA acetyltransferase, mitochondrial precursor | 2 | 4 | 45.2 |
| BLMH | bleomycin hydrolase | 2 | 4 | 52.5 |
| CCDC58 | coiled-coil domain-containing protein 58 isoform 1 | 2 | 4 | 16.6 |
| MSMP | prostate-associated microseminoprotein precursor | 2 | 4 | 15 |
| STMN1 | stathmin isoform b | 2 | 4 | 19.8 |
| TMED7-TICAM2 | TRAM adaptor with GOLD domain isoform 1 precursor | 2 | 4 | 46.1 |
| RPS3 | 40S ribosomal protein S3 isoform 2 | 2 | 4 | 28.5 |
| CIRBP | cold-inducible RNA-binding protein isoform 3 | 2 | 4 | 31.9 |
| CALM2 | calmodulin isoform 1 | 2 | 4 | 22.2 |
| MDH1 | malate dehydrogenase, peroxisomal isoform MDH1x | 2 | 4 | 38.6 |
| ANXA4 | annexin A4 isoform a | 2 | 4 | 36.1 |
| BSG | basigin isoform 1 precursor | 2 | 4 | 42.2 |
| CCT6A | T-complex protein 1 subunit zeta isoform a | 2 | 4 | 58 |
| CNN3 | calponin-3 isoform 1 | 2 | 4 | 36.4 |
| EIF6 | eukaryotic translation initiation factor 6 isoform a | 2 | 4 | 26.6 |
| RPN1 | dolichyl-diphosphooligosaccharide--protein glycosyltransferase subunit 1 precursor | 2 | 4 | 68.5 |
| SNRPA1 | U2 small nuclear ribonucleoprotein A' | 2 | 4 | 28.4 |
| DSP | desmoplakin isoform I | 2 | 4 | 331.6 |
| FKBP2 | peptidyl-prolyl cis-trans isomerase FKBP2 precursor | 2 | 4 | 15.6 |
| RAB7A | ras-related protein Rab-7a | 2 | 4 | 23.5 |
| PDCD5 | programmed cell death protein 5 | 2 | 4 | 14.3 |
| TLN1 | talin-1 | 2 | 4 | 269.6 |
| PPIH | peptidyl-prolyl cis-trans isomerase H | 2 | 4 | 19.2 |
| PROCR | endothelial protein C receptor precursor | 2 | 4 | 26.7 |
| NPTN | neuroplastin isoform b precursor | 2 | 4 | 44.4 |
| PPP2R1A | serine/threonine-protein phosphatase 2A 65 kDa regulatory subunit A alpha isoform | 2 | 4 | 65.3 |
| RALY | RNA-binding protein Raly isoform 1 | 2 | 4 | 32.4 |
| APMAP | adipocyte plasma membrane-associated protein | 2 | 4 | 46.5 |
| RPS18 | 40S ribosomal protein S18 | 2 | 4 | 17.7 |
| CYB5B | cytochrome b5 type B | 2 | 4 | 16.7 |
| CDC42 | cell division control protein 42 homolog isoform 2 | 2 | 4 | 21.3 |
| RPLP0 | 60S acidic ribosomal protein P0 | 2 | 4 | 34.3 |
| MTPN | Myotrophin | 2 | 4 | 12.9 |
| PDCD10 | PREDICTED: programmed cell death protein 10 isoform X1 | 2 | 4 | 24.7 |
| IMPDH2 | PREDICTED: inosine-5'-monophosphate dehydrogenase 2 isoform X1 | 2 | 4 | 66.1 |
| PUF60 | PREDICTED: poly(U)-binding-splicing factor PUF60 isoform X1 | 2 | 4 | 64 |
| RMDN1 | PREDICTED: regulator of microtubule dynamics protein 1 isoform X1 | 2 | 4 | 44 |
| A2ML1 | PREDICTED: alpha-2-macroglobulin-like protein 1 isoform X1 | 2 | 4 | 162.5 |
| ARHGDIA | PREDICTED: rho GDP-dissociation inhibitor 1 isoform X1 | 2 | 4 | 27.5 |
| DSTN | PREDICTED: destrin isoform X1 | 2 | 4 | 18.5 |
| TKT | PREDICTED: transketolase isoform X1 | 2 | 4 | 68.8 |
| CAPN1 | PREDICTED: calpain-1 catalytic subunit isoform X1 | 2 | 4 | 81.8 |
| G3BP1 | PREDICTED: ras GTPase-activating protein-binding protein 1 isoform X1 | 2 | 4 | 52.1 |
| CPNE3 | PREDICTED: copine-3 isoform X1 | 2 | 4 | 60.1 |
| RAN | PREDICTED: GTP-binding nuclear protein Ran isoform X1 | 2 | 4 | 25.3 |
| QARS | glutamine--tRNA ligase isoform a | 3 | 3 | 87.7 |
| HNRNPA0 | heterogeneous nuclear ribonucleoprotein A0 | 3 | 3 | 30.8 |
| NENF | neudesin precursor | 3 | 3 | 18.8 |
| NOMO2 | nodal modulator 2 isoform 1 precursor | 2 | 3 | 139.4 |
| SLC3A2 | 4F2 cell-surface antigen heavy chain isoform b | 2 | 3 | 68.1 |
| HNRNPR | heterogeneous nuclear ribonucleoprotein R isoform 1 | 2 | 3 | 71.2 |
| RBBP7 | histone-binding protein RBBP7 isoform 1 | 2 | 3 | 52.3 |
| ACADVL | very long-chain specific acyl-CoA dehydrogenase, mitochondrial isoform 3 | 2 | 3 | 72.9 |
| TPT1 | translationally-controlled tumor protein isoform 1 | 2 | 3 | 22.6 |
| TBCA | tubulin-specific chaperone A isoform 1 | 2 | 3 | 15.8 |
| CAPN2 | calpain-2 catalytic subunit isoform 1 | 2 | 3 | 80 |
| CTSC | dipeptidyl peptidase 1 isoform a preproprotein | 2 | 3 | 51.8 |
| PSMA3 | proteasome subunit alpha type-3 isoform 1 | 2 | 3 | 28.4 |
| TXN | thioredoxin isoform 1 | 2 | 3 | 11.7 |
| ZPR1 | zinc finger protein ZPR1 isoform 1 | 2 | 3 | 50.9 |
| ASNA1 | ATPase ASNA1 | 2 | 3 | 38.8 |
| ETF1 | eukaryotic peptide chain release factor subunit 1 isoform 1 | 2 | 3 | 49 |
| GSTO1 | glutathione S-transferase omega-1 isoform 1 | 2 | 3 | 27.5 |
| PGLS | 6-phosphogluconolactonase | 2 | 3 | 27.5 |
| SND1 | staphylococcal nuclease domain-containing protein 1 | 2 | 3 | 101.9 |
| CACYBP | calcyclin-binding protein isoform 1 | 2 | 3 | 26.2 |
| PGM5 | phosphoglucomutase-like protein 5 | 2 | 3 | 62.2 |
| TMX1 | thioredoxin-related transmembrane protein 1 precursor | 2 | 3 | 31.8 |
| S100A13 | PREDICTED: protein S100-A13 isoform X1 | 2 | 3 | 11.5 |
| NAP1L1 | PREDICTED: nucleosome assembly protein 1-like 1 isoform X1 | 2 | 3 | 45.3 |
| PRDX4 | PREDICTED: peroxiredoxin-4 isoform X1 | 2 | 3 | 39.7 |
| COL1A2 | collagen alpha-2(I) chain precursor | 2 | 2 | 129.2 |
| GLRX3 | glutaredoxin-3 isoform 1 | 2 | 2 | 37.4 |
| DDB1 | DNA damage-binding protein 1 | 2 | 2 | 126.9 |
| XPO1 | exportin-1 | 2 | 2 | 123.3 |
| COX5A | cytochrome c oxidase subunit 5A, mitochondrial precursor | 2 | 2 | 16.8 |
| LCN2 | neutrophil gelatinase-associated lipocalin precursor | 2 | 2 | 22.6 |
| TRIM28 | transcription intermediary factor 1-beta | 2 | 2 | 88.5 |
| HNRNPM | heterogeneous nuclear ribonucleoprotein M isoform a | 2 | 2 | 77.5 |
| VCP | transitional endoplasmic reticulum ATPase | 2 | 2 | 89.3 |
| DYNC1LI1 | cytoplasmic dynein 1 light intermediate chain 1 | 2 | 2 | 56.5 |
| ENOPH1 | enolase-phosphatase E1 isoform 1 | 2 | 2 | 28.9 |
| ADGRE5 | CD97 antigen isoform 1 preproprotein | 2 | 2 | 91.8 |
| CAPZB | PREDICTED: F-actin-capping protein subunit beta isoform X1 | 2 | 2 | 34.5 |
| NUDC | PREDICTED: nuclear migration protein nudC isoform X1 | 2 | 2 | 45.2 |
| SDHA | PREDICTED: succinate dehydrogenase [ubiquinone] flavoprotein subunit, mitochondrial isoform X1 | 2 | 2 | 85.6 |
| VDAC1 | PREDICTED: voltage-dependent anion-selective channel protein 1 isoform X1 | 2 | 2 | 30.8 |
